# *HSP90* and *HSP70* Families in *Lateolabrax maculatus*: Genome-Wide Identification, Molecular Characterization, and Expression Profiles in Response to Various Environmental Stressors

**DOI:** 10.3389/fphys.2021.784803

**Published:** 2021-11-22

**Authors:** Yalong Sun, Haishen Wen, Yuan Tian, Xuebin Mao, Xiurong Li, Junjie Li, Yanbo Hu, Yang Liu, Jifang Li, Yun Li

**Affiliations:** ^1^Key Laboratory of Mariculture, Ocean University of China, Ministry of Education, Qingdao, China; ^2^Quality and Safety Center of Agricultural and Livestock Products, Bayannaoer, China

**Keywords:** *HSP90*, *HSP70*, spotted seabass, environmental stresses, gene expression, transcriptome

## Abstract

Heat shock proteins (HSPs) are a large class of highly conserved chaperons, which play important roles in response to elevated temperature and other environmental stressors. In the present study, 5 *HSP90* genes and 17 *HSP70* genes were systematically characterized in spotted seabass (*Lateolabrax maculatus*). The evolutionary footprint of *HSP* genes was revealed via the analysis of phylogeny, chromosome location, and gene copy numbers. In addition, the gene structure features and the putative distribution of heat shock elements (HSEs) and hypoxia response elements (HREs) in the promoter regions were analyzed. The protein-protein interaction (PPI) network analyses results indicated the potential transcriptional regulation between the heat shock factor 1 (*HSF1*) and *HSPs* and a wide range of interactions among *HSPs*. Furthermore, quantitative (q)PCR was performed to detect the expression profiles of *HSP90* and *HSP70* genes in gill, liver, and muscle tissues after heat stress, meanwhile, the expression patterns in gills under alkalinity and hypoxia stresses were determined by analyzing RNA-Seq datasets. Results showed that after heat stress, most of the examined *HSP* genes were significantly upregulated in a tissue-specific and time-dependent manners, and *hsp90aa1.1, hsp90aa1.2*, *hsp70.1*, and *hsp70.2* were the most intense responsive genes in all three tissues. In response to alkalinity stress, 11 out of 13 significantly regulated *HSP* genes exhibited suppressed expression patterns. Alternatively, among the 12 hypoxia-responsive-expressed *HSP* genes, 7 genes showed induced expressions, while *hsp90aa1.2*, *hsp70.1*, and *hsp70.2* had more significant upregulated changes after hypoxic challenge. Our findings provide the essential basis for further functional studies of *HSP* genes in response to abiotic stresses in spotted seabass.

## Introduction

Heat shock proteins (HSPs) are a large group of molecular chaperones with highly conserved sequence structures, which are classified into five families, namely, *HSP90*, *HSP70*, *HSP60/HSP10*, *HSP40*, and small heat shock protein (*sHSP*) based on their molecular weights (MWs). The expressions of HSPs are constitutive as housekeeping genes in unstressed cells or induced significantly by abiotic and biotic stressors (Feder and Hofmann, 1999). Since the first discovery of HSPs in the salivary gland cells of the fruit fly (*Drosophila busckii*) (Ritossa, 1962; Tissiéres et al., 1974), the identification and function studies of HSPs have been widely reported in a broad range of vertebrates and invertebrates. For aquaculture species, *HSP90* and *HSP70* are the most widely studied HSPs in the very large HSP family, which are essential for the maintenance of protein homeostasis and cellular recovery after various environmental stresses (Hangzo et al., 2016; Umam et al., 2016; Gupta et al., 2020).

Heat shock proteins 90 and *HSP70* genes are evolutionarily conserved molecular chaperones that are important for the folding and regulation of a variety of cellular proteins in an adenosine triphosphate (ATP)-dependent manner (Sib Sankar et al., 2014). In humans, 6 HSP90 genes (HSP90AA1, HSP90AA2, HSP90AB1, HSP90B1, TRAP1, and HSP90N) and 17 HSP70 genes (HSPA6, HSPA7, HSPA4L, HSPA9B, HSPA4, HSPA1L, HSPA1A, HSPA1B, HSPA5, HSPA12A, HSPA14, HSPA8, HYOU1, HSPH1, HSPA2, HSPA12B, and STCH) were identified (Chen et al., 2005; Brocchieri et al., 2008). Functional HSP90 is a homodimer with each monomer composed of three major domains: an ATP binding N-terminal domain (N-domain) (Prodromou et al., 1997), a protein-binding middle domain (M-domain), and a C-terminal domain (C-domain) that interacts with co-chaperones (Street et al., 2011; Xie et al., 2015). The three domains that are linked via flexible linkers can homodimerize to create an *HSP90* active unit, which has a conserved function in the reconfiguration of abnormally folded proteins to their normal state through ATP hydrolysis and structural rearrangement (Zuehlke and Johnson, 2010). *HSP70s* are the most highly conserved HSPs, which contain two major domains: an N-domain that controls the interaction with the client protein, and a C-domain, which is the substrate-binding domain that identifies the hydrophobic regions in the client during its initial folding stage. The two domains are also connected by a flexible linker (Bukau et al., 2006; Gupta et al., 2020). When *HSP70* is not bound to a cellular protein, its ATPase activity is lower than average. The co-chaperon, J-domain protein family, assists client protein to bind with *HSP70*, vitalizing its ATPase activity, and facilitating protein folding and transport (Gupta et al., 2020). *HSP70* transforms to its apo form by liberating ADP from it after the J-domain protein leaves this, which makes the N-domain engage ATP, leading to native conformation of client protein to release (Schlecht et al., 2011; Gupta et al., 2020). In addition to carrying out chaperone activities independently, *HSP90* and *HSP70* were identified to interact directly and collaborate to assist in numerous cellular progresses (Wegele et al., 2006; Zuehlke and Johnson, 2010; Doyle et al., 2019; Gupta et al., 2020).

Fish are frequently subjected to varieties of environmental pressures, such as high or low temperature, low oxygen, osmotic stress, or other challenges caused by poor water quality. Owing to the important roles of *HSP90* and *HSP70* genes in response to abiotic stress factors, it is of great significance to understand their molecular structures and functional mechanism in teleosts. As the increasing number of available genomic resources, more and more *HSP90* genes and *HSP70* genes have been discovered in teleost species, and their potential involvement under environmental stresses have been investigated by examining the gene expression changes. For example, a total of 8 *HSP90* and 16 *HSP70* genes were identified in rainbow trout (*Oncorhynchus mykiss*), respectively (Ma et al., 2020; Ma and Luo, 2020). The significantly differential expressions of six *HSP90* genes and four *HSP70* genes in the liver, and six *HSP90* genes and six *HSP70* genes in the head kidney were reported after heat stress treatment, indicating they may take part in heat stress response in rainbow trout (Ma et al., 2020). In large yellow croaker (*Larimichthys crocea*), systematic gene characterization has been conducted for the *HSP70* family and 17 genes have been identified. By examining RNA-seq data, six *HSP70* genes were significantly upregulated or downregulated in the liver after cold or heat challenge, indicating their involvement in defending against thermal stresses (Xu et al., 2018). Moreover, the complete gene sets of *HSP90* and/or *HSP70* have been identified for channel catfish (*Ictalurus punctatus*) and mudskipper (*Boleophthalmus pectinirostris*) (Song et al., 2014; Xie et al., 2015; Deng et al., 2019). In addition, differentially expression of *HSP90* and *HSP70* genes after the hypoxia stress (Shi et al., 2020), hypotonic stress (Umam et al., 2016), and ammonia stress (Hangzo et al., 2016) has been demonstrated in fishes. These studies suggest that *HSP90* and *HSP70* genes are potential modulators participating in the heat and other abiotic stress, and the functions of *HSPs* genes may vary among different teleost species.

Spotted seabass (*Lateolabrax maculatus; L. maculatus*) is an economically important aquaculture fish species in China of its high yield, high nutritional value, and pleasant taste (Liu et al., 2020; Yu et al., 2020) with the annual production exceeding 190,000 tons in recent years (China Fishery Statistical Yearbook, 2021; Li et al., 2021). Nevertheless, it is facing various environmental threatens, such as heat stress caused by global warming, salinity stress derived from tidal water flow, and the oxidative stress along with the development of intensive aquaculture. These stressors lead to a reduction of the fitness of fish or even death, severely affecting its economic benefits. However, little is known about the roles of HSP molecular chaperones under environmental stresses in spotted seabass. Therefore, in this study, systematic identification and characterization of *HSP90* and *HSP70* genes were conducted in spotted seabass. Through phylogenetic and homology analyses, their annotation was determined, and their evolutionary relationship was clarified. Furthermore, the presence of conserved *Cis-*regulatory elements in the promoter region and the potential protein interaction network dominated by HSP90 and HSP70 were predicted. To provide insight into the function of the *HSP90* and *HSP70* genes of spotted seabass in response to various environmental stresses, their expression profiles in target tissues were detected after being challenged by different abiotic stress conditions, such as heat, alkalinity, and hypoxia stresses. Our results will reveal the molecular characteristics of the *HSP90* and *HSP70* gene family in spotted seabass and provide a theoretical basis for the in-depth study of their biological functions under abiotic stresses.

## Materials and Methods

### Identification of Heat Shock Protein Genes in Spotted Seabass

To identify *HSP90* and *HSP70* genes in *L. maculatus*, the reference genome (BioProject: PRJNA408177) and transcriptomic databases (SRR4409341, SRR4409397) were searched using TBLASTN based on the query sequences of *HSP90* and *HSP70* genes from human, mouse (*Mus musculus*), chicken (*Gallus gallus*), tropical clawed frog (*Xenopus tropicalis*), zebrafish, spotted gar (*Lepisosteus oculatus*), Mexican tetra (*Astyanax mexicanus*), tongue sole (*Cynoglossus semilaevis*), Atlantic salmon (*Salmo salar*), and channel catfish (*Ictalurus punctatus*) retrieved from the National Center for Biotechnology Information (NCBI)^1^ and previous research (Song et al., 2014) (The query sequence identifiers are listed in Supplementary Table 1), with a cutoff E-value of 1e^––5^. The open reading frames (ORFs) were predicted, and the retrieved sequences were translated by ORF Finder.^2^ Predicted ORFs were validated by BLASTP against NCBI non-redundant protein database.

The gene copy numbers of *HSP90* and *HSP70* family were compared based on the genome databases of spotted seabass and several selected vertebrates, such as human, mouse, chicken, zebrafish, channel catfish, Japanese medaka (*Oryzias latipes*), fugu (*Takifugu rubripes*), spotted gar, large yellow croaker, and Nile tilapia (*Oreochromis niloticus*). The chromosomal location of each *HSP* gene was shown according to the coordinates of each *HSP* gene on the spotted seabass genome.

### Phylogenetic and Syntenic Analysis

Phylogenetic analysis was conducted using the amino acid sequences of *HSP90* and *HSP70* genes from several representative vertebrates retrieved from NCBI and Ensembl^3^ databases, such as human, mouse, chicken, zebrafish, spotted gar, Atlantic salmon, channel catfish, Nile tilapia, large yellow croaker, fugu, and Japanese medaka (Supplementary Table 1). After conducting multiple alignments of *HSP* sequences by MUSCLE with default parameters, the phylogenetic tree was constructed using MEGA 7 with the neighbor-joining method. Jones-Taylor-Thornton (JTT) + invariant sites (I) + gamma distribution for modeling rate heterogeneity (G) model was selected and bootstrapping with 1,000 replications was conducted to evaluate the phylogenetic tree. The tree was further modified using Interactive Tree of Life 6 (iTOL 6^4^).

The syntenic analysis was performed to provide additional evidence for the annotation of the duplicated *HSP90* and *HSP70* genes. The neighboring genes of *hsp90aa1, hspa8a, hyou1, hsp70.1*, and *hsp70.2* were identified from spotted seabass reference genome and further compared with the genome regions of selected representative vertebrates, which were obtained from NCBI and Genomicus databases 100.01^5^.

### Sequence Analysis of *HSP90* and *HSP70* Genes

Protein characteristics (MW and isoelectric points, pIs) of *HSP* genes in spotted seabass were predicted by the Prot Param tool^6^ using the deduced amino acids. The homologous domain was surveyed by the SMART 7.0 program^7^ and the NCBI conserved domain database.^8^ The gene structures of *HSPs* were obtained from the *L. maculatus* reference genome database and were visualized using the GSDS 2.0 software.^9^ The conserved motifs of *HSPs* were observed with the MEME online tool^10^ and were displayed using TBtools 1.068.

For the prediction of heat shock elements (HSEs) and hypoxia response elements (HREs), 2-kb upstream regions from the transcription start site (TSS) of each *HSP* gene were extracted from the spotted seabass reference genome and analyzed using JASPAR 2018 server.^11^

### Protein-Protein Interaction Network Prediction

The Protein-Protein Interaction (PPI) relationships of the *HSPs* were predicted by constructing a zebrafish association model using STRING 11.0 software^12^ with deduced amino acid sequences of spotted seabass.

### Heat Stress Experiment

The heat stress experiment was conducted at Shuangying Aquaculture Company, Dongying, Shandong, China. Sixty spotted seabass juveniles (body length: 13.33 ± 0.24 cm, body weight: 38.96 ± 2.01 g) were collected and acclimated for 2 weeks in a tank [5 m × 5 m × 1.5 m (L × W × H)]. During acclimation period, the experimental conditions were kept constant, such as water temperature (25.0 ± 1.0^*o*^C), pH (7.5 ± 0.4), salinity (31 ± 1.0 ppt), and dissolved oxygen (DO) (7.0 ± 0.5 mg/L).

After acclimation, 60 individuals were transferred to three tanks at the density of 20 per tank. The temperature was settled as 25^*o*^C, and fish were acclimated for 48 h. After that, the water temperature was increased at a constant rate of 1^*o*^C/h until it reached 32°C, and thereafter, the temperature was maintained. Three individuals per tank were sampled at each time point, such as 0 h (H0), 10 h (3 h after heat stress, H3), 19 h (12 h after heat stress, H12), and 31 h (24 h after heat stress, H24) after heat stress (Supplementary Figure 1). Sampled fish were anesthetized with tricaine methanesulfonate (MS-222, 200 mg/L), and gill, liver, and muscle tissues were dissected and flash-frozen in liquid nitrogen for RNA extraction.

### Expression of *HSP90* and *HSP70* Genes Under Heat Stress by Quantitative Real-Time PCR Analysis

Total RNA of gill, liver, and muscle tissues in heat stress experiment was extracted using TRIzol reagent (Invitrogen, Waltham, CA, United States) according to the instructions of manufacturer. RNA concentration and integrity were measured by using the Biodropsis BD-1000 nucleic acid analyzer (Beijing Oriental Science & Technology Development Ltd., Beijing, China) and 1.5% agarose gel electrophoresis, respectively. cDNA was synthesized using PrimeScriptTM RT reagent Kit (Takara, Otsu, Japan) following the protocol of the manufacturer. qPCR was performed using the StepOne Plus Real-Time polymerase chain reaction (PCR) system (Applied Biosystems, Foster City, CA, United States) to detect the expression patterns of *HSP90* and *HSP70* mRNA. Primers of *HSP90* and *HSP70* genes were designed by Primer 6 software (Table 1). *18S* rRNA was used as the internal control to correct the qPCR veracity, and the samples were run in triplicate (Wang et al., 2018). The 20 μl qPCR reaction volume included 2 μl template cDNA, 0.4 μl each forward/reverse primer, 10 μl 2 × ChamQ SYBR Color qPCR Master Mix, and 7.2 μl of nuclease-free water. The qPCR amplification was carried out using the following procedures: 95^*o*^C for 30 s and 40 cycles of 95^*o*^C for 5 s, 55^*o*^C for 30 s, and 72^*o*^C for 30 s. The relative mRNA expression levels of *HSP90* and *HSP70* genes were calculated according to the 2^–ΔΔCT^ method.

**TABLE 1:** Primers used for quantitative real-time PCR (qPCR).

**Gene**	**Primer sequence (5′-3′)**
*hsp90aa1.1*	F: CAATGATGACGAGCAG R: AGGGTAGCCAATGAAC
*hsp90aa1.2*	F: GCTGAACAAAACCAAACC R: AAAGAGTAGGGCACGGA
*hsp90ab1*	F: TCGTGGAGACGCTCAGACAG R: GTAGATGCGGTTGGAGTGGG
*hsp90b1*	F: TGGTTGCCAGTCAGTACG R: GATGTTTGGGATTGATTTCT
*trap1*	F: TGGTTCCAAGGCATTTT R: GCAGCACTATTTTCGTTCC
*hspa8a.1*	F: CAAGAAGGACATCAGCGACAAC R: CAAGGTGCCACGGAAGAGG
*hspa8a.2*	F: AGGACATCAGCGACAACAAGAG R: CAAGGTGCCACGGAAGAGG
*hspa8b*	F: CTGCTGCTGCTATTGCTTACG R: CCTCTTGTTGTCGCTGATGTC
*hsc70*	F: CAAGACCTGCTTCTGCTGGA R: TGGTCATGGCTCTCTCACCT
*hspa1b*	F: TCTCAGAGGCAAGCAACAAAGG R: TCAAAGATGCCGTCCTCAATGG
*hsp70.1*	F: GGACGCCGACAAATACAAAGC R: CGATGGTCTGGTCACACTTCTC
*hsp70.2*	F: CCTCATCCAGGTCTACG R: CTGCTCATCCTCGCTAA
*hspa5*	F: GACATTGGTGGTGGCCAGAT R: CAGGAACAGTGACCACAGCA
*hspa9*	F: TTGGCATTGACCTGGGAACC R: TGTGAAGGCGATGACTGAAGG
*hspa13*	F: CTCAACCTCACCCTCCAA R: CGGCTAACACGGTCTCAA
*hyou1*	F: TCGGGTGGAGTCGGTCTTTG R: TGTCCTCGTCCTTGCCATCC
*hspa4a*	F: CGGCATGTTCAGTGTATCAAGC R: GGCTGGTCGTTCTTCTTCTCC
*hspa4b*	F: CACAGTCCAAGGGTTCAAGAGG R: CACAACACAGTCAGCCACAGG
*hspa4l*	F: AAGGCAGAGGACCAGACC R: TGACTTTGGGCTTGCTTCC
*hspa14*	F: GGCTGAAGTCGTGGCTAA R: GGTAATGACCGCATCTGTG
*hspa12a*	F: TCGGATGCGATGGCTAAT R: CGTGGTTGGAGTCTTCTGA
*hspa12b*	F: CGACTACTACAAGCGACACAGG R: CACCAACCAGGAAGAGGAAGC
*18S*	F: GGGTCCGAAGCGTTTACT R: TCACCTCTAGCGGCACAA

ANOVA and Duncan’s multiple tests were applied to assess the means of the relative mRNA expression level using SPSS 26.0 software. The differences were considered as statistically significant when the *P*-value < 0.05. The graphs were depicted by the software of GraphPad Prism.8.0.2.

### Expression Profiles of *HSP90* and *HSP70* Genes Following Alkalinity and Hypoxia Treatments by Analysis of RNA-Seq Datasets

To investigate the responsiveness of *HSP90* and *HSP70* genes to different abiotic stress conditions, RNA-Seq datasets generated by our previous challenge experiments of alkalinity and hypoxia were used to determine the gene expression pattern.

Briefly, for the alkalinity challenge experiment, 1-year-old spotted seabass individuals (body weight: 140.32 ± 2.56 g) were firstly acclimated in fresh water (pH: 7.8 ± 0.4) for 30 days. Carbonate-alkalinity solution was prepared by adding NaHCO_3_ (12.8 mmol/L) and Na_2_CO_3_ (2.6 mmol/L) to fresh water and aerated for 24 h before the experiment. The carbonate alkalinity (mmol/L) was monitored every day during the exposure period using acidimetric titrations. After acclimation, 45 spotted seabass individuals were immediately transferred to three replicated 100 L square tanks with alkaline water (carbonate alkalinity: 18 ± 0.2 mmol/L), and the dissolved oxygen concentration, temperature, and pH were maintained at 7.1 ± 0.4 mg/L, 22 ± 1^*o*^C and pH: 9.0 ± 0.2, respectively. The fish were not fed during the stress experiment. Gill tissues of three fish individuals in each tank were sampled at several time points, such as 0, 12, 24, and 72 h after alkalinity stress. The samples were frozen in liquid nitrogen and stored at − 80^*o*^C for RNA extraction. RNA of the three individuals from the same tank was pooled as one sample, and 12 sequencing libraries (3 replicated samples × 4 time points) were generated. Total 150 bp paired-end reads (PRJNA611641) were obtained by the Illumina HiSeq X Ten platform.

For hypoxic treatment experiment, spotted seabass individuals (body weight: 178.25 ± 18.56 g) were randomly divided into two groups: normoxic group (6.89 ± 0.25mg/L) and hypoxic group (1.1 ± 0.14 mg O_2_/L) in triplicate tanks at the density of 20 fish per tank. The threshold DO level was set as 1.1 ± 0.14 mg O_2_/L, which was determined by preliminary experiments. The oxygen level of the hypoxia group was reduced to 1.1 ± 0.14 mg/L by bubbling nitrogen gas for 30 min. During the experiment period, the dissolved oxygen levels were maintained constant by a mixture of air and nitrogen gas. Gill tissues of three fish per tank (a total of nine individuals for each time point) were sampled at 0, 3, 6, and 12 h after the hypoxia challenge. A total of 12 sequencing libraries (3 replicated samples X 4 time points) were generated. Total 150 bp paired-end reads (unpublished data) were generated using the Illumina HiSeq 4000 platform.

For each RNA-seq project, the high-quality clean reads from each library were generated using Trimmomatic 0.36 and then mapped to the reference genome of spotted seabass (PRJNA408177) using HISAT 2 (-p 4 –dta -t –phred33) with no mismatch. The mapped reads from alignments were counted and then normalized to determine expected number of Fragment Per Kilobase of transcript sequence per Million base pairs sequenced (FPKM). Differential expression in fold change of each *HSP* gene was determined based on the ratio of the expression value. Differential expression statistical analysis was performed using the DESeq 2 R package with *P*-value < 0.05.

## Results

### Identification and Characteristics of *HSP90* and *HSP70* Genes in Spotted Seabass

A total of 5 *HSP90* (*hsp90aa1.1*, *hsp90aa1.2*, *hsp90ab1*, *hsp90b1*, and *trap1*) and 17 *HSP70* (hspa*8a.1*, *hspa8a.2*, *hspa8b*, *hsc70*, *hspa1b*, *hsp70.1*, *hsp70.2*, *hspa5*, *hspa9*, *hspa13*, *hyou1*, *hspa4a*, *hspa4b*, *hspa4l*, *hspa14*, *hspa12a*, and *hspa12b*) genes were identified in spotted seabass. Their detailed information is summarized in Table 2. The ORF of *HSP90* genes was ranged from 1,944 to 2,241 bp, encoding protein lengths different from 647 to 744 amino acid (aa). Their predicted MW was varied from 74.22 to 85.31 kDa, and pI were from 4.65 to 5.99. *HSP70* genes contained ORFs varying from 1,329 to 2,982 bp in length and encoded proteins with 442 to 993 aa. MWs of *HSP70* genes were distinct from 48.10 to 110.39 kDa, and pIs were from 4.95 to 7.22. All *HSPs* sequences of spotted seabass had been submitted to GenBank databases, and their accession numbers are listed in Table 2.

**TABLE 2:** Summary of sequence characteristics of *HSP90* and *HSP70* genes in spotted seabass.

	**Clade**	**Gene name**	**ORF length (bp)**	**Predicted protein length (aa)**	**MW (kDa)**	**Isoelectric point (pI)**	**Domain**	**Domain location (aa)**	**NCBI accession number**
*HSP90*	***HSP90AA1***	*hsp90aa1.1*	1944	647	74.22	4.91	HATPase_c	28–182	MN646875
		*hsp90aa1.2*	2127	708	81.98	5.18	HATPase_c	36–190	MN646876
	***HSP90AB1***	*hsp90ab1*	1989	663	76.44	5.38	HATPase_c	11–165	MN646877
	***HSP90B1***	*hsp90b1*	2241	744	85.31	4.65	HATPase_c	65–224	MN646878
	***TRAP1***	*trap1*	2148	715	81.20	5.99	HATPase_c	119–272	MN646879
*HSP70*	***HSPA8***	*hspa8a.1*	1683	560	61.48	5.18	HSPA1-2_6-8-like_NBD	1–295	MN646861
		*hspa8a.2*	2982	993	110.19	6.81	HSPA1-2_6-8-like_NBD	412–723	MN646860
		*hspa8b*	1758	585	64.40	5.43	HSPA1-2_6-8-like_NBD	7–319	MN646863
	***HSPA1***	*hsc70*	1950	649	71.00	5.18	HSPA1-2_6-8-like_NBD	6–381	MN646862
		*hspa1b*	1920	639	70.62	5.54	HSPA1-2_6-8-like_NBD	8–383	MN646857
		*hsp70.1*	1866	621	68.28	5.70	HSPA1-2_6-8-like_NBD	8–383	MN646858
		*hsp70.2*	1920	639	70.10	5.42	HSPA1-2_6-8-like_NBD	8–383	MN646859
	***HSPA5***	*hspa5*	1965	654	72.23	4.95	HSPA5-like_NBD	26–401	MN646872
	***HSPA9***	*hspa9*	2043	680	73.68	6.43	HSPA9-like_NBD	52–428	MN646873
	***HSPA13***	*hspa13*	1329	442	48.10	5.92	HSPA13-like_NBD	12–434	MN646864
	***HYOU1***	*hyou1*	2961	986	110.39	5.00	HYOU1-like_NBD	29–415	MN646874
	***HSPA4***	*hspa4a*	2517	838	93.98	5.23	HSPA4_NBD	2–384	MN646867
		*hspa4b*	2532	843	94.50	5.06	HSPA4_NBD	2–384	MN646865
		*hspa4l*	2499	832	93.26	5.35	HSPA4-like_NBD	2–384	MN646866
	***HSPA14***	*hspa14*	1521	506	54.66	5.72	HSPA14-like_NBD	2–376	MN646868
	***HSPA12***	*hspa12a*	2016	671	75.17	6.72	HSPA12A-like_NBD	55–521	MN646869
		*hspa12b*	2058	685	76.15	7.22	HSPA12B-like_NBD	55–525	MN646870

*ORF, open reading frame; MW, molecular weights; NCBI, the National Center for Biotechnology Information.*

### Phylogenetic and Syntenic Analysis

**FIGURE 1:**
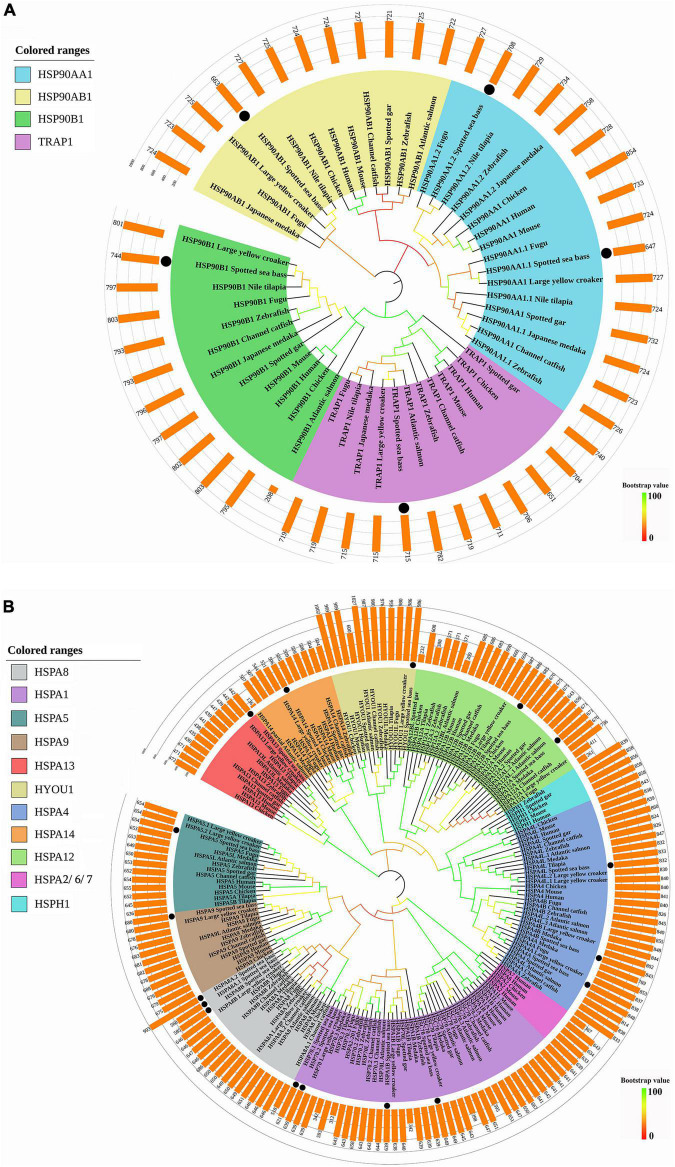
Phylogenetic analyses of **(A)** *HSP90* and **(B)** *HSP70* genes. The phylogenetic tree was constructed by the deduced amino acid sequences with 1,000 bootstrap replications in MEGA 7. Different sizes of predicted amino acids of these genes were represented by the bars upon the phylogenetic tree. The black dots represented the genes of spotted seabass.

As shown in Figure 1A, *HSP90* genes of spotted seabass were clustered with respective counterparts and four clades were generated, namely, HSP90AA1, HSP90AB1, HSP90B1, and TRAP1, which were consistent with their annotation. Two copies of *hsp90aa1* (*hsp90aa1.1* and *hsp90aa1.2*) in spotted seabass were orthologous to teleost *hsp90aa1.1* and *hsp90aa1.2*, respectively.

All the *HSP70* genes of spotted seabass were well distributed into distinct clades and grouped with homologous genes of selected species, supported by strong bootstrap values (Figure 1B). Eleven clades were generated, such as HSPA8, HSPA1, HSPA5, HSPA9, HSPA13, HYOU1, HSPA4, HSPA14, HSPA12, HSPA2/6/7, and HSPH1, and *HSP70* genes of spotted seabass were distributed in nine of them (HSPA8, HSPA1, HSPA5, HSPA9, HSPA13, HYOU1, HSPA4, HSPA14, and HSPA12). *HSPH1* genes were only discovered for selected tetrapods and a few teleosts, meanwhile *hspa2/6/7* was specific to tetrapods. The annotation of five genes (*hspa5*, *hspa9*, *hspa13*, *hyou1*, and *hspa14*) with a single copy and duplicated genes, such as *hspa12* (*hspa12a* and *hspa12b*) and *hspa4* (*hspa4a*, *hspa4b*, and *hspa4l*), could be well supported by the phylogenetic relationships, but it is hard to distinguish and name the remaining *HSP70* genes with duplicated copies solely by phylogeny.

**FIGURE 2:**
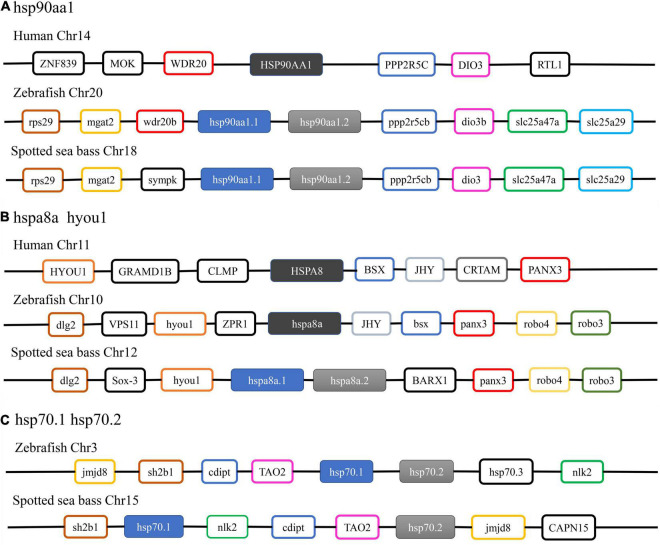
Syntenic analysis of **(A)** *hsp90aa1*; **(B)** *hspa8a*; **(C)** *hsp70.1* and *hsp70.2* among human, zebrafish, and spotted seabass.

Thus, syntenic analysis was performed to provide additional evidence for the annotation of three duplicated *HSP* genes in spotted seabass (Figure 2). For *hsp90aa1*, the *hsp90aa1.1* and *hsp90aa1.2* of zebrafish and spotted seabass were tandem located, with highly similar upstream neighbor genes like *rps29* and *mgat2* and downstream genes as *ppp2r5cb*, *dio3b*, *slc25a47a* and *slc25a29*. A unique gene copy of HSP90AA1 was found for human, and its neighbor genes, such as WDR20, PPP2R5C, and DIO3, were conserved with tested teleosts (Figure 2A). For all tested three species, *hspa8*/*hspa8a* and *hyou1* were located at the same chromosome. Duplicated gene copies of *hspa8a* (*hspa8a.1* and *hspa8a.2*) were identified for spotted seabass, alternatively, human and zebrafish harbored single *hspa8* and *hspa8a* genes, respectively. Neighboring regions of *hspa8a* were more conserved between zebrafish and spotted seabass in comparison with human (Figure 2B). The synteny analyses of *hsp70.1* and *hsp70.2* were only conducted in zebrafish and spotted seabass due to the absence of *hsp70.1* and *hsp70.2* genes in human. As shown in Figure 2C, *hsp70.1* and *hsp70.2* were tandem duplicated in the zebrafish genome, while *hsp70.1* and *hsp70.2* in spotted seabass were separated by *nlk2*, *cdipt*, and *TAO2.* However, they shared a relatively conserved genomic neighborhood.

Taken together, the phylogenetic and syntenic analysis results not only well support the annotation and nomenclature of the *HSP90* and *HSP70* genes in spotted seabass but also indicate that these *HSP* gene families were highly conserved in the evolution.

### Chromosome Distribution

**FIGURE 3:**
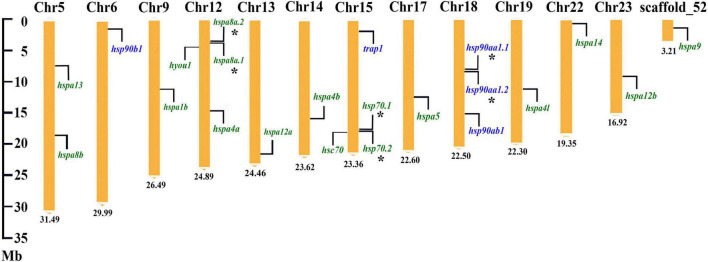
Chromosome location of the *HSP90* and *HSP70* genes in spotted seabass. The *HSP90* and *HSP70* genes were indicated by the purple and green fonts, respectively. Asterisks (*) indicated tandem duplicated genes. The number at the bottom represented the length of the chromosomes or scaffold.

For the chromosomal location of target *HSP* genes, 5 *HSP90* genes were distributed on three chromosomes, and 17 *HSP70* genes were located on 10 chromosomes and one un-anchored scaffold, respectively (Figure 3). In detail, for the *HSP90* family, *hsp90ab1* and tandem duplicated *hsp90aa1.1* and *hsp90aa1.2* were located on chr18, meanwhile, *hsp90b1* and *trap1* were located on chr6 and chr15, respectively. For the *HSP70* family, chr12 possessed the largest gene number of *HSP70s*, that four genes, such as the tandem arranged *hspa8a.2* and *hspa8a.1*, and *hyou1* and *hspa4a*, were located on it. The tandem duplicated *hsp70.1* and *hsp70.2*, and *hsc70* were positioned on chr15. Additionally, two *HSP70* genes, *hspa13* and *hspa8b*, were located on chr5. For the rest nine *HSP70* genes (*hspa1b*, *hspa12a*, *hspa4b*, *hspa5*, *hspa4l*, *hspa14*, *hspa12b*, and *hspa9*), they were distributed on the different chromosomes and scaffold fragment (Chr9, Chr13, Chr14, Chr17, Chr19, Chr22, Chr23, and scaffold_52), respectively.

### Gene Copy Numbers of *HSP90* and *HSP70* Genes

Copy numbers of the *HSP90* and *HSP70* genes in representative vertebrates are summarized in Table 3. In general, the number of *HSP90* genes was relatively conserved across a broad spectrum of vertebrate species from mammals to fishes, that the total gene numbers of *HSP90* were slightly varied from 4 to 5 among different species. Only one copy of each gene was present except for that *hsp90aa1*. *hsp90aa1* was duplicated in several detected teleosts, such as zebrafish, Japanese medaka, fugu, Nile tilapia, and spotted seabass, but a single copy was detected in tetrapods.

**TABLE 3:** Copy numbers of *HSP90* and *HSP70* genes among several representative vertebrate species.

	**Name**	**human**	**mouse**	**Chicken**	**zebrafish**	**channel catfish**	**Japanese medaka**	**fugu**	**spotted gar**	**large yellow croaker**	**Nile tilapia**	**spotted sea bass**
	*hsp90aa1*	1	1	1	2	1	2	2	1	1	2	2
	*hsp90ab1*	1	1	1	1	1	1	1	1	1	1	1
*HSP90*	*hsp90b1*	1	1	1	1	1	1	1	1	1	1	1
	*trap1*	1	1	1	1	1	1	1	1	1	1	1
	*Total*	4	4	4	5	4	5	5	4	4	5	5
	*hspa1*	3	3	0	5	3	4	4	2	3	3	4
	*hspa2*	1	1	1	0	0	0	0	0	0	0	0
	*hspa4*	2	2	2	3	3	3	2	2	4	1	3
	*hspa5*	1	1	1	1	1	1	1	1	2	2	1
	*hspa6*	1	0	0	0	0	0	0	0	0	0	0
	*hspa7*	1	0	0	0	0	0	0	0	0	0	0
*HSP70*	*hspa8*	1	1	1	2	3	1	1	0	2	2	3
	*hspa9*	1	1	1	1	1	1	1	1	1	1	1
	*hspa12*	2	2	2	3	2	2	2	3	2	2	2
	*hspa13*	1	1	1	1	1	1	1	1	1	1	1
	*hspa14*	1	1	1	1	1	1	1	1	1	1	1
	*hsph1*	1	1	1	1	0	0	0	1	0	0	0
	*hyou1*	1	1	1	1	1	1	1	1	1	1	1
	Total	17	15	12	19	16	15	14	13	17	14	17

Gene copy number of the *HSP70* family ranged from 13 to 19 among these detected species. In accordance with the phylogenetic analysis results, *hspa6* and *hspa7* genes were only discovered in human, and *hspa2* was only present in higher vertebrates, such as human, mouse, and chicken. Four *HSP70* genes were single-copy in all selected species, such as *hspa9*, *hspa13*, *hspa14*, and *hyou1*. Multiple gene copies were found in five *HSP70* genes of teleosts, such as *hspa1*, *hspa4*, *hspa5*, *hspa8*, and *hspa12*. Among them, *hspa1* harbored the highest copy numbers in most detected species, and *hspa5* and *hspa8* were only duplicated in teleost species (Table 3).

### Exon-Intron Structure and Motif Analysis

**FIGURE 4:**
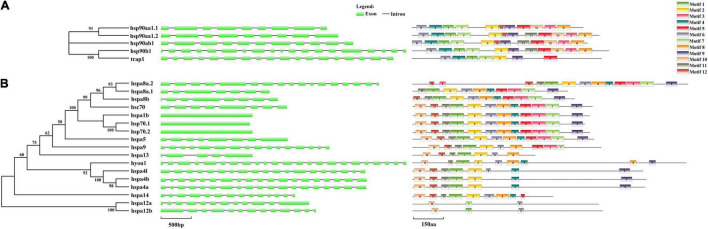
Exon-intron structure and motif analyses of **(A)** *HSP90* and **(B)** *HSP70* in spotted seabass. Exons were shown by green rectangles and introns were indicated by black lines. Lengths of exons and introns are displayed proportionally according to the scale.

Exon-intron structures and motif analysis were employed to investigate the conservatism and diversity of gene structures in *HSP90* and *HSP70* genes (Figure 4). The intron numbers of the *HSP90* genes varied from 8 to 18. Among them, *hsp90aa1.1* and *hsp90aa1.2* harbored highly similar exon-intron structures (Figure 4A). Contrastingly, the intron numbers of the *HSP70* genes were remarkably varied from 0 to 22 (Figure 4B). According to the number of introns, *HSP70* genes could be divided into two modes: mode 1 contained no intron, such as *hspa1b*, *hsp70.1*, and *hsp70.2*, and the rest genes belonged to mode 2, which possessed multiple introns, with intron numbers varied from 4 to 22. In general, the paralogous gene pairs shared similar exon-intron structures (Figure 4B).

For the motif analysis results, the motif numbers among *HSP90* genes varied from 7 to 12, whereas all *HSP90* members harbored seven conservative motifs, such as motif 1, motif 2, motif 4, motif 5, motif 6, motif 7, and motif 9 (Figure 4A). For the *HSP70* family, most genes shared five conservative motifs, namely, motif 1, motif 2, motif 6, motif 10, and motif 11. Among them, *hsc70, hspa1b, hsp70.1, hsp70.2*, and *hspa5* shared highly similar motif pattern, although belonging to two distinct exon-intron structure modes (with or without intron). Moreover, the paralogous gene groups, such as *hspa4* genes (*hspa4a*, *hspa4b*, and *hspa4l*) and *hspa12* genes (*hspa12a* and *hspa12b*), shared conserved motif pattern (Figure 4B). The length of each *HSP90* and *HSP70* motifs was varied from 21 to 50 and 29 to 50 amino acids, respectively.

### Putative Heat Shock Elements and Hypoxia Response Elements in the Promoter Regions of the Heat Shock Protein Genes

**FIGURE 5:**
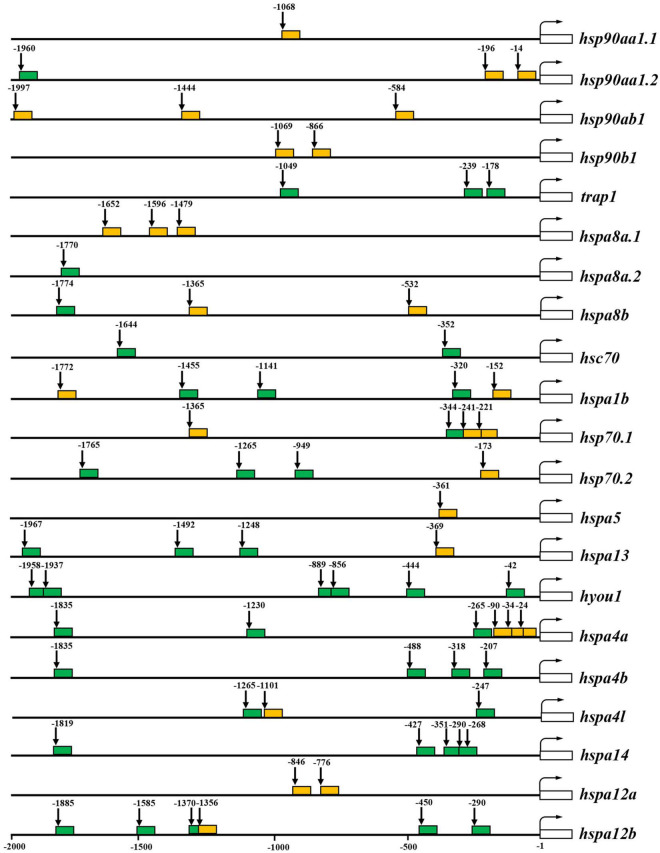
Putative heat shock elements (HSEs) and hypoxia response elements (HREs) in 2 kb upstream regions from the transcription start site (TSS) of *HSP90* and *HSP70* genes. The predicted HSEs and HREs were shown with orange and green filled boxes, respectively. The numbers indicated spacing (in base pairs) between elements and TSS. The white open box indicates the transcribed sequence. The bent arrow indicates the TSS.

As *cis*-regulatory elements, HSEs and HREs are the master regulators which bind to HSFs and hypoxia-inducible factors (HIFs) to mediate the transcriptional response of their target genes, respectively. Therefore, the distributions of these elements in the promoter regions of *HSP90* and *HSP70* genes were predicted for spotted seabass. As shown in Figure 5, 28 HSEs and 43 HREs were predicted in the selected promoter regions of *HSPs* genes. The HSE numbers were various among different *HSP* genes ranging from 0 to 3. In the 2 kb upstream region from the TSS, three HSEs were detected for *hsp90ab1*, *hspa8a.1*, *hsp70.1*, and *hspa4a*, two HSEs were identified for *hsp90aa1.2*, *hsp90b1*, *hspa8b*, *hspa1b*, and *hspa12a* genes, meanwhile only one HSE was existed in *hsp90aa1.1*, *hsp70.2*, *hspa5*, *hspa13*, *hspa4l*, and *hspa12b* (Figure 5).

For the prediction results of HREs, 0–6 HRE numbers were discovered in the 2 kb upstream of TSS of target *HSP* genes. *Hyou1* harbored six HREs, meanwhile, *hspa14* and *hspa12b* harbored five HREs. Four HREs were identified in *hspa4b*, three HREs were detected for *trap1*, *hspa1b*, *hsp70.2*, *hspa13*, and *hspa4a*. *Hsc70* and *hspa4l* contained two HREs. Unique HRE position has existed in 2 kb upstream from the TSS of *hsp90aa1.2*, *hspa8a.2*, *hspa8b*, and *hsp70.1* (Figure 5).

It can be deduced from the above result that six *HSP* genes (*hsp90aa1.1*, *hspab1*, *hsp90b1*, *hspa5*, *hspa8a.1*, and *hspa12a*) contained only HSE in their promoter regions, meanwhile six genes (*trap1*, *hspa4b*, *hspa8a.2*, *hspa14*, *hsc70*, and *hyou1*) only possessed HREs. Alternatively, nine genes, namely, *hsp90aa1.2*, *hspa8b*, *hspa1b*, *hsp70.1*, *hsp70.2*, *hspa13*, *hspa4a*, *hspa4l*, and *hspa12b*, harbored both HSE and HRE in their promoter regions (Figure 5).

### Protein-Protein Interaction Network Analysis

As the transcription of HSPs is mainly regulated by heat shock factor 1 [HSF1 (Buckley and Hofmann, 2001], the PPI networks of HSPs and HSF1 in spotted seabass were constructed using STRING database based on the orthologs in zebrafish. The results indicated that 22 HSPs of spotted seabass were closely associated with 21 known zebrafish HSPs participated in the interaction network (Supplementary Figure 2 and Supplementary Table 2). As expected, extensive interactions were observed between the HSF1 and HSPs, which was revealed by a direct link representing “transcriptional regulation” in Supplementary Figure 2.

Additionally, HSP70s interacted with HSP70s, and HSP90s also be related to HSP90s, respectively. For example, HSPA4A, HSPA5, and HSPA9 showed a wide range of interactions (reaction, binding, and catalysis) with other HSP70 family proteins. Meanwhile, HSP90AA1.1, HSP90AA1.2, and HSP90AB1 proteins were connected by blue lines, suggesting their “binding” relationship (Supplementary Figure 2). Furthermore, strong interaction relationships were commonly found between HSP70 and HSP90 proteins, for instance, HSP90AA1.1, HSP90AA1.2, and HSP90AB1, were closely connected to HSPA4A, implying that the former may play a negative role in transcriptional regulation of the latter (Supplementary Figure 2).

### Expression Patterns of *HSP90* and *HSP70* Genes in Response to Heat Stress by Quantitative PCR Analysis

**FIGURE 6:**
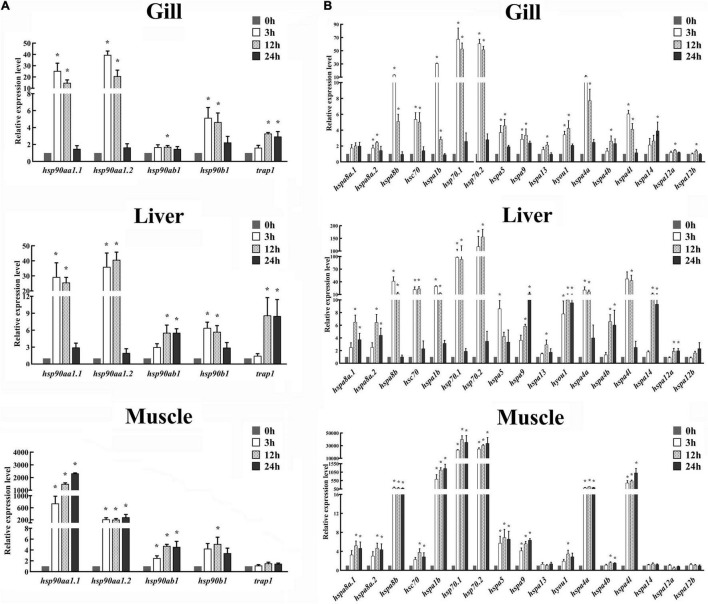
Expression patterns of **(A)** *HSP90* and **(B)** *HSP70* genes in the gill, liver, and muscle of spotted seabass at 0, 3, 12, and 24 h after heat stress. The mRNA expression levels were determined by qPCR analysis. 18S rRNA was used as an internal control. Gene expressions were presented as mean ± SE. Asterisk (*) represented the significant differences (*P* < 0.05). HSP, heat shock proteins.

The expression patterns of *HSP90* and *HSP70* genes in gill, liver, and muscle tissues of spotted seabass were systematically examined at 0, 3, 12, and 24 h after heat stress. Results showed that except for *trap1* in the muscle, all *HSP90* genes were significantly induced after heat stress in tissue- and time-dependent manner (Figure 6A). Among them, *hsp90aa1.1* and *hsp90aa1.2* exhibited the biggest expression changes in all three detected tissues, with their expression levels remarkably increased to at least 14.8-fold. The highest expression level of *hsp90aa1.1* and *hsp90aa1.2* appeared at 24 h in the muscle, with a 2,330-fold and 285-fold increase compared with 0 h, respectively. In gill, three genes (*hsp90aa1.1*, *hsp90aa1.2*, and *hsp90b1*) exhibited similar expression patterns after heat stress, which were significantly upregulated at 3 and 12 h and then reduced their expression after 24 h. The results were paralleled with the observation of their expression patterns in the liver (Figure 6A). In contrast, in muscle, the induced expressions of *hsp90aa1.1*, *hsp90aa1.2*, and *hsp90ab1* genes were appeared for 3 h, maintaining the high expression levels until the end of the challenge experiment. A significant heat stress-responsive expressions were also detected for *hsp90ab1* and *trap1* in the gill and liver and *hsp90b1* in the muscle, with their upregulated expression levels appeared since 12 h after heat stress treatment (Figure 6A).

Similarity, significant expression variations were commonly identified for several *HSP70* genes in three target tissues after heat stress (Figure 6B). In gill tissue, excerpt for *hspa8a.1*, all *HSP70* genes displayed significant differential expression patterns after heat treatment. Among them, highly heat stress-induced expressions in gill were discovered for *hspa8b, hspa1b*, *hsp70.1, hsp70.2*, and *hspa4a* genes, with the maximum expression values exceeded 10-fold compared with those under normal conditions (0 h). Their expression was dramatically induced at the initial tested phase of post-heat stress (3 h) and gradually scaled down during subsequent time points (Figure 6B). In the liver, remarkable upregulations were detected for all *HSP70* genes with one exception that the expression change of *hspa12b* was not statistically significant (Figure 6B). The highest expression variations were discovered for *hspa8b, hsc70, hspa1b, hsp70.1, hsp70.2, hspa4l*, and *hspa4a*, which showed similar expression profile after heat stress treatment, that their expressions were dramatically induction at 3 h, remaining highly until 12 h, and then decreased to their normal expression level at 24 h (Figure 6B). The other heat stress-responsive *HSP70* genes, namely, *hspa8a.1*, *hspa8a.2*, *hspa9*, *hspa4b*, and *hspa14*, exhibited dramatically induced expressions at 12 and 24 h. The expression of *hyou1* was highly upregulated at all three tested time points after heat treatment (Figure 6B). For muscle tissues, the expressions of 13 *HSP70* genes, such as *hspa8a.1*, *hspa8a.2*, *hspa8b*, *hsc70*, *hspa1b*, *hsp70.1*, *hsp70.2*, *hspa5*, *hspa9*, *hyou1*, *hspa4a*, *hspa4b*, and *hspa4l*, were significantly increased after heat stress. Among them, the upregulated expression levels of *hspa1b, hsp70.1, hsp70.2*, and *hspa4l* were extremely significant, exceeding hundreds of times than their normal expression values. Unlike the expression pattern in gill and liver, most differential expressed *HSP70*s, such as *hspa8b*, *hspa1b*, *hsp70.1*, *hsp70.2*, *hspa5*, *hspa9*, *hspa4a*, and *hspa4l*, displayed upregulation at all time points after heat treatment (Figure 6B). It was worth noting that *hsp70.1* and *hsp70.2* were the most intense responsive genes in all three tissues after heat stress treatment, suggesting the two genes may play significant roles in response to heat stress in spotted seabass.

### Expression Patterns of *HSP90* and *HSP70* Genes in Response to Alkalinity Challenge and Hypoxia Stress by Examining RNA-Seq Data Sets

**FIGURE 7:**
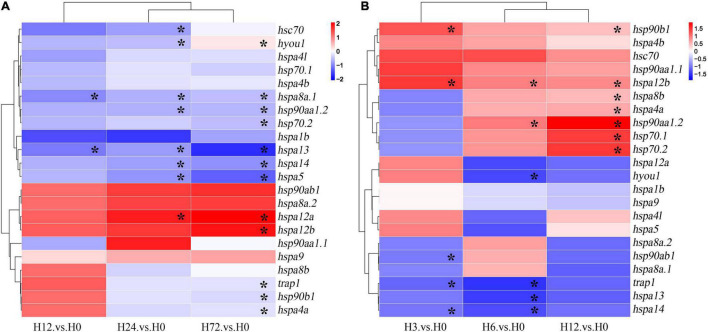
Expression profiles of *HSP90* and *HSP70* genes in gills of spotted seabass in response to **(A)** alkalinity and **(B)** hypoxia stresses. The abundance of mRNA was determined by analyzing the RNA-Seq data. A color scale in the heatmaps represented the fold change values. Asterisk (*) indicated the significant differences (*P* < 0.05). HSP, heat shock proteins.

As revealed by RNA-Seq analysis, after alkalinity stress, the *HSP* genes in gills of spotted seabass exhibited dynamic time-dependent expression pattern, and the number of differential expressed genes increased as the alkalinity treating time was prolonged (Figure 7A and Supplementary Table 3). A total of 11 genes, namely, *hsp90aa1.2*, *hsp90b1*, *trap1*, *hsp70.2*, *hspa8a.1*, *hsc70*, *hspa13*, *hspa4a*, *hspa14*, *hspa5*, and *hyou1*, showed downregulated expression profile (fold change: − 1.23 to − 3.06, *P* < 0.05). In detail, downregulated expressions of *hspa8a.1* and *hspa13* were found since 12 h after alkalinity treatment, a trend that continues until the final experimental time point (72 h). The other genes showed suppressed expression levels starting from the later time points (24 or 72 h). The highest expression changes of *HSP* genes during the alkalinity challenge experimental period were detected for *hspa13*, with fold change values nearly double in comparison with 0 h. Conversely, only two *HSP* genes (*hspa12a* and *hspa12b*) showed upregulated expressions after alkalinity stress (*P* < 0.05).

After the hypoxia stress, a time-dependent expression profile was also detected for *HSP* genes in gill, however, the expression trend was distinct from those under alkalinity stress. As shown in Figure 7B and Supplementary Table 4, a total of 12 genes (*hsp90aa1.2*, *hspab1*, *hsp90b1*, *trap1*, *hsp70.1*, *hsp70.2*, *hspa8b*, *hspa13*, *hspa4a*, *hspa14*, *hspa12b*, and *hyou1*) in the gill of spotted seabass were changed significantly after the hypoxia challenge (*P* < 0.05). In general, five genes, namely, *hsp90ab1*, *trap1*, *hyou1*, *hspa13*, and *hspa14*, exhibited downregulated trends (fold change: − 1.23 to − 1.47, *P* < 0.05), conversely, more genes, such as *hsp90aa1.2*, *hsp90b1*, *hsp70.1*, *hsp70.2*, *hspa8b*, *hspa4a*, and *hspa12b*, showed remarkably induced expression at 12 h during low-oxygen treatment experiment (fold change: 1.50–3.97, *P* < 0.05). Among the hypoxia-responsive expressed genes, *hsp90aa1.2*, *hsp70.1*, and *hsp70.2* had more significant upregulated changes than other *HSPs*, with expression changes at 12 h exceeding threefold (Figure 7B and Supplementary Table 4).

## Discussion

In this study, we performed a systemic analysis of the *HSP90* and *HSP70* gene families of spotted seabass, revealing the gene features by phylogenic, syntenic, and gene structure analysis and investigated their potential involvement in response to environmental stresses by examining the gene expression patterns. Our results may lay an important basis for further evolutionary and functional studies of HSPs in fish species.

A total of 5 *HSP90* (*hsp90aa1.1*, *hsp90aa1.2*, *hsp90ab1*, *hsp90b1*, and *trap1*) and 17 *HSP70* genes (*hspa8a.1*, *hspa8a.2*, *hspa8b*, *hsc70*, *hspa1b*, *hsp70.1*, *hsp70.2*, *hspa5*, *hspa9*, *hspa13*, *hyou1*, *hspa4a*, *hspa4b*, *hspa4l*, *hspa14*, *hspa12a*, and *hspa12b*) were identified in the spotted seabass by searching genome and several transcriptomic databases. The annotation of identified HSP genes was validated by phylogenetic and syntenic analysis, and the evolution and expansion/contraction of *HSP90* and *HSP70* genes were revealed through the analysis of the phylogenetic relationship and gene copy numbers. These were evidences for the evolutionary conservativeness of *HSP90* and *HSP70* genes. The HATPase_c and HSPA/HYOU1_(like_) NBD domains were detected in all *HSP90* or *HSP70* genes of spotted seabass, respectively (Table 2), which was considered to be a classification standard for unknown HSP targets (Mao et al., 2006; Wu et al., 2016). For the *HSP90* family, in comparison with tetrapods that each gene harbored the unique copy, duplicated genes of *hsp90aa1* existed in several teleosts genomes (Table 3). Based on the conserved linear arrangement of genes between zebrafish and spotted seabass (Figures 2A, 3), we named the two *hsp90aa1* copies as *hsp90aa1.1* and *hsp90aa1.2* accordingly. Their tandem arrangement in genome indicated that the additional gene copy may have arisen through local gene tandem duplication or called small-scale duplication (SSD) events instead of whole-genome duplication (WGD). As in the *HSP70* family, *hspa8a.1* and *hspa8a.2* were tandem located at the same chromosome (Chr12), which may also derive from SSD event (Figures 2B, 3). Alternatively, as shown in the phylogenetic tree of the *HSP70* family (Figure 1B), after the divergence between teleosts and tetrapods, *hspa4* in teleosts replicates into two independent genes (*hspa4a* and *hspa4b*), which was a newer duplication than *hspa4l*. These three gene copies were situated at different chromosomes, which indicated the paralogs may arise from a teleost-specific WGD event (Figure 3). In contrast, *hspa2* did not exist in all selected fishes, while *hspa6* and *hspa7* were only discovered in human, suggesting that these genes were gained in higher vertebrate during evolution (Xie et al., 2015).

To obtain insight into the structural diversity of the spotted seabass *HSP* genes, the intron–exon organization was analyzed (Figure 4). The introns numbers of the *HSP90* genes varied from 8 to 18, while *HSP70* genes harbored introns ranging from 4 to 22 or had no intron (Figure 4B). Previous studies have demonstrated that different exon-intron structures of *HSP* genes were closely associated with their unique biological functions (Huang et al., 2018; Xu et al., 2018). For example, depending on the intron-exon structure, members of the *HSP70* genes have two regulatory patterns at the transcriptional level. Firstly, the constitutively expressed *HSPs*, usually contain multiple introns, presenting in the cell under all conditions to help folding the newly translated proteins. Secondly, the inducible members of *HSP70* genes, typically lacking introns, are significantly expressed under stress conditions (Xu et al., 2018). In spotted seabass, *hspa1b*, *hsp70.1*, and *hsp70.2* were identified as the most responsive genes in all three tested tissues after heat stress, which was probably due to their intron-less structure (Figures 4, 6B). It has been proved that the intron-less genes might be produced by the loss of multiple introns from the intron-rich gene by retrotransposition during gene family diversification (Wang et al., 2019).

The expression of *HSP* is mainly regulated by the binding of HSF1 to HSEs in the promoter region of the HSP genes. Under non-stressful conditions, HSF1 exists as an inactive monomer. However, when exposed to heat or other types of cellular stress, HSF1 is converted into trimeric form, binding to promoters of downstream target genes, whereby initiating the transcription (Kmiecik et al., 2020). Previous studies have shown that distance from the TSS to the HSEs may alter the strength of the *HSP70* promoter, and closer spacing facilitates more rapid transcription (Tian et al., 2010). In spotted seabass, the HSEs in the promoter regions of *hsp90aa1.2*, *hspa1b*, *hsp70.1*, *hsp70.2*, and *hspa4a* were relatively closer to the TSS (Figure 5), which may lead to the strong responsive expression levels after heat stress (Figure 6). On the contrary, no HSE was detected in several genes (Figure 5), indicating that alternative regulatory mechanisms might underlie these genes mediated by HSR (Huang et al., 2018). Besides, the HIF-1 complex has been reported to activate the transcription of some *HSP* genes containing the HREs when adapting to cellular stress imposed by hypoxia (Huang et al., 2009; Gogate et al., 2012). In spotted seabass, the predicted HRE numbers and distance from the TSS to the HREs were also varied among different *HSP* genes (Figure 5), which were speculated to be related to their different degree of response to hypoxic stress, and the detailed mechanism need to be investigated further.

To explore the interaction of gene-encoded proteins of the *HSP* genes, we predicted the PPI network using the deduced protein sequence of *HSP90* and *HSP70*. The results showed that extensive connections existed among the predicted *HSP90* and *HSP70* family proteins (Supplementary Figure 2), which was in line with the expectation as the two chaperone machines participate together in countless cellular processes (Luengo et al., 2019).

Although the previous study has reported the expressions of one *HSP90* and one *HSP70* gene in spotted seabass during thermal stress experiment (Shin et al., 2018), the distinguish and participation of the full gene sets of the two gene families in response to heat stress in this species has not been systematically analyzed. In our study, the heat stress-responsive expression changes of *HSP90* and *HSP70* gene family in gill, liver, and muscle, which was reported as primary tissues responsible for heat stress in teleost (Giri et al., 2014; Purohit et al., 2014; Shi et al., 2015; Peng et al., 2016; Shin et al., 2018; Xu et al., 2018), have been investigated thoroughly. As the results showed, *hsp90aa1.1* and *hsp90aa1.2* in the *HSP90* family and *hspa8b, hsc70, hspa1b, hsp70.1, hsp70.2, hspa4a*, and *hsp4l* in the *HSP70* family displayed remarkable heat-inducible expression patterns in two or three tested tissues of spotted seabass (Figure 6B). Although only *HSP70* family were examined, in line with our findings, five *HSP70* genes (*hspa4a, hsc70, hspa5.1, hspa8b*, and *hsp70*) were significantly upregulated in liver of large yellow croaker under heat treatment, and *hsp70* and *hspa8b* were the most inducible genes which were considered as the strictly heat-inducible *HSP70* genes (Xu et al., 2018). As we stated above, the strong induction of these genes during heat shock may be generated due to the lack of introns allows efficient transcription (e.g., *hspa1b*, *hsp70.1*, and *hsp70.2*), or the close distance from the HSEs to the TSS facilitates rapid transcription (e.g., *hsp90aa1.2*, *hspa1b*, *hsp70.1*, *hsp70.2*, and *hspa4a*).

In addition, we examined the potential involvement of *HSP90* and *HSP70* genes of spotted seabass in response to other two important abiotic stressors, alkalinity and hypoxia, by analyzing the transcriptomic datasets. In contrast to the overall highly inducible expression of *HSP* genes after heat stress, a significant portion of genes exhibited significantly suppressed expression levels under alkalinity and/or hypoxia challenge (Figure 7). For example, compared with the dramatic upregulated expressions of *hsp70.1*, *hsp70.2*, and *hspa1b* under heat stress, mild downregulated expression of *hsp70.2* was found at 72 h in gills after alkalinity treatment (Figure 7A, *P* < 0.05). Besides, *hsp70.1* and *hspa1b* exhibited a downregulated expression trend under alkalinity treatment, in spite of being not statistically significant (Figure 7A). The distinct expression patterns indicated that different responding mechanisms might underlie these *HSP* genes mediated by specific environmental stressors. Similar results have been also reported for *Litopenaeus vannamei*, that by testing the response sensitivity and intensity of *HSP*s to different environmental stresses, *hsp70* gene was considered as the biomarker indicating thermal stress, but not suitable for acting as an indicator of pH stress (Qian et al., 2012). The downregulated expression of *HSP70* genes has also been reported in the brain of the large yellow croaker under hypoxia stress (Ao et al., 2015), and in the gills of *Boleophthalmus pectinirostris* under the oxidative stress caused by high environmental ammonia (Deng et al., 2019). Moreover, the significant upregulated expressions of *hsp90aa1.2*, *hsp70.1* and *hsp70.2* after hypoxic challenge were identified in spotted seabass (Figure 7B), and similar expression profiles have also been reported for Indian Catfish (*Clarias batrachus*) and Amur sturgeon (*Acipenser schrenckii*) (Ni et al., 2014; Mohindra et al., 2015). Although the mechanism underlying the stress-regulated expression levels of *HSP* genes has not yet been fully elucidated, the *HSPs* with differential expression patterns may play their respective roles in protection and survival under stress conditions.

## Conclusion

In this study, the entire *HSP90* and *HSP70* gene families were systematically characterized in spotted seabass. Phylogenetic, syntenic, and gene copy numbers analyses provided sufficient evidences for the annotation and evolutionary footprint of these genes. Gene structures and *cis-*regulatory elements including HSEs and HREs were further characterized, which not only showed that genes with closer homology relationships had similar structures and conserved motifs, but also give a hint to explain the significant stress-regulated expression patterns of several *HSP* genes. Moreover, the potential involvement of *HSPs* in response to heat stress was determined by the qPCR experiment and dynamic time-dependent expression pattern after alkalinity and hypoxic challenge were revealed through the analysis of RNA-Seq data. Our findings provided a comprehensive overview of *HSP90* and *HSP70* families in spotted seabass and laid the basis for a better understanding of the physiological function of *HSP90* and *HSP70* genes in response to abiotic stress.

## Data Availability Statement

The original contributions presented in the study are included in the article/Supplementary Material, further inquiries can be directed to the corresponding author/s.

## Ethics Statement

The animal study was reviewed and approved by Animal Research and Ethics Committee of Ocean University of China (Permit Number: 20141201).

## Author Contributions

YS, YT, JuL, XM, and YH performed bioinformatics analysis. YuL and JiL provided funding support. YuL and YS conceived the study. YS, YaL, XL, and XM performed stress experiments. HW and YuL administrated the project. All authors read and approved the submitted version.

## Conflict of Interest

The authors declare that the research was conducted in the absence of any commercial or financial relationships that could be construed as a potential conflict of interest.

## Publisher’s Note

All claims expressed in this article are solely those of the authors and do not necessarily represent those of their affiliated organizations, or those of the publisher, the editors and the reviewers. Any product that may be evaluated in this article, or claim that may be made by its manufacturer, is not guaranteed or endorsed by the publisher.

## Footnotes

1
http://www.ncbi.nlm.nih.gov/

2
https://www.ncbi.nlm.nih.gov/gorf/gorf.html

3
http://www.ensembl.org

4
http://itol.embl.de/

5
https://www.genomicus.bio.ens.psl.eu/genomicus-100.01/cgi-bin/search.pl

6
http://www.expasy.ch/tools/protparam.html

7
http://smart.embl.de/

8
https://www.ncbi.nlm.nih.gov/Structure/cdd/wrpsb.cgi

9
http://gsds.cbi.pku.edu.cn

10
http://meme.nbcr.net/meme/

11
http://jaspar.genereg.net/

12
https://string-db.org/

## Supplementary Material

Supplementary Figure 1 Schematic diagram of heat stress experiment. Spotted seabass individuals were acclimated for 48 h at ambient temperature (25^*o*^C) before heat stress treatment. Water temperature was elevated at a constant rate of 1^*o*^C/h until 32°C. Fish individuals were sampled at four time points including 0 h (H0), 10 h (3 h after heat stress, H3), 19 h (12 h after heat stress, H12) and 31 h (24 h after heat stress, H24), respectively.

Supplementary Figure 2 Predicted protein-protein interaction network of HSF1 and HSPs in spotted seabass. This network was predicted by the online STRING database. The balls represented the gene nodes, and the connecting lines and arrowheads indicated the interactions. HSP, heat shock proteins; HSF, heat shock factor.
