# Supplementary figures and images for: HSP90 and HSP70 Families in Lateolabrax maculatus: Genome-Wide Identification, Molecular Characterization, and Expression Profiles in Response to Various Environmental Stressors
Source: Front Physiol. 2021 Nov 22;12:784803. doi: 10.3389/fphys.2021.784803 (PMC8646100; doi:10.3389/fphys.2021.784803)

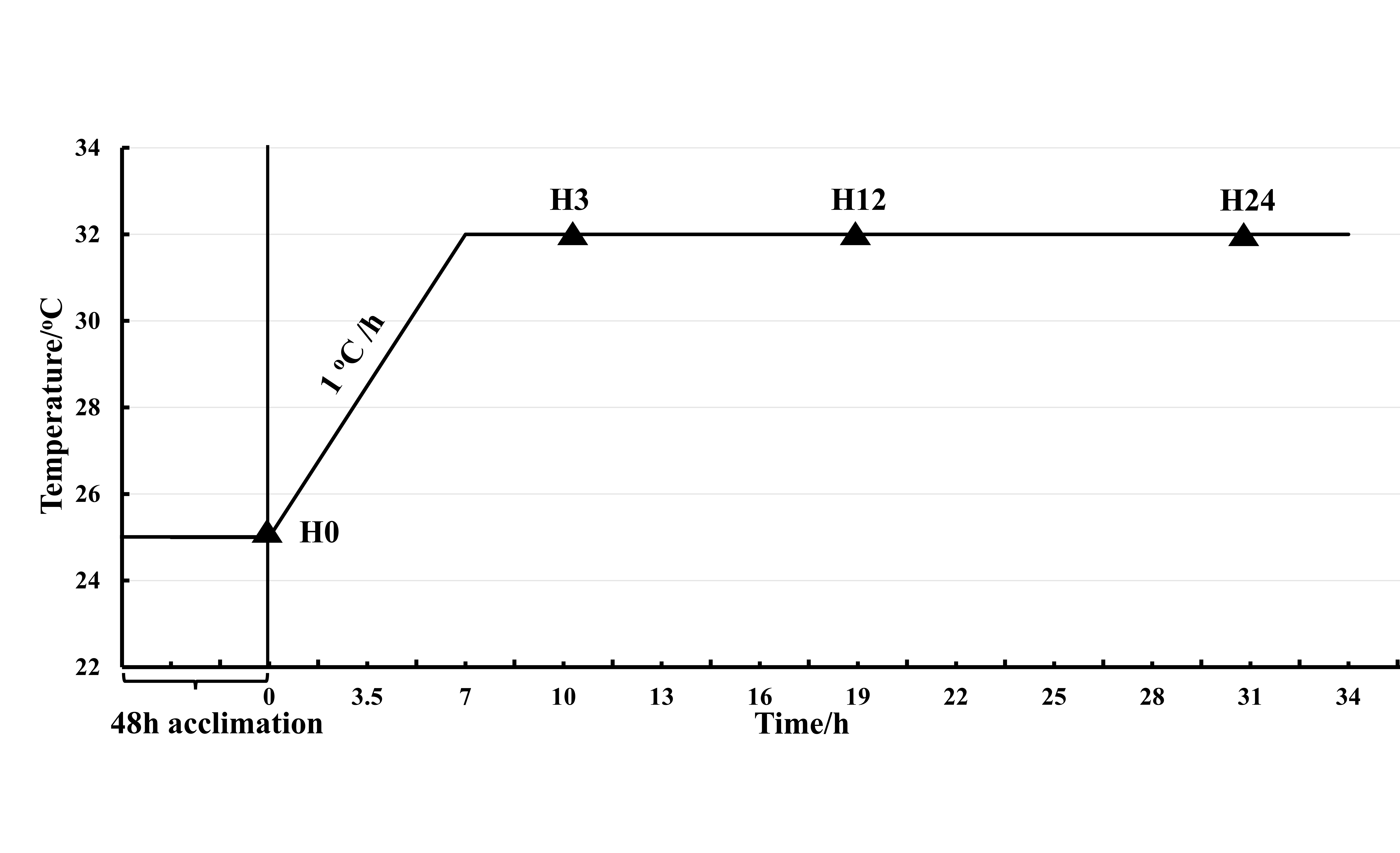

Supplement: Supplementary Figure 1 — Schematic diagram of heat stress experiment. Spotted seabass individuals were acclimated for 48 h at ambient temperature (25oC) before heat stress treatment. Water temperature was elevated at a constant rate of 1oC/h until 32°C. Fish individuals were sampled at four time points including 0 h (H0), 10 h (3 h after heat stress, H3), 19 h (12 h after heat stress, H12) and 31 h (24 h after heat stress, H24), respectively. [file Image_1.JPEG]

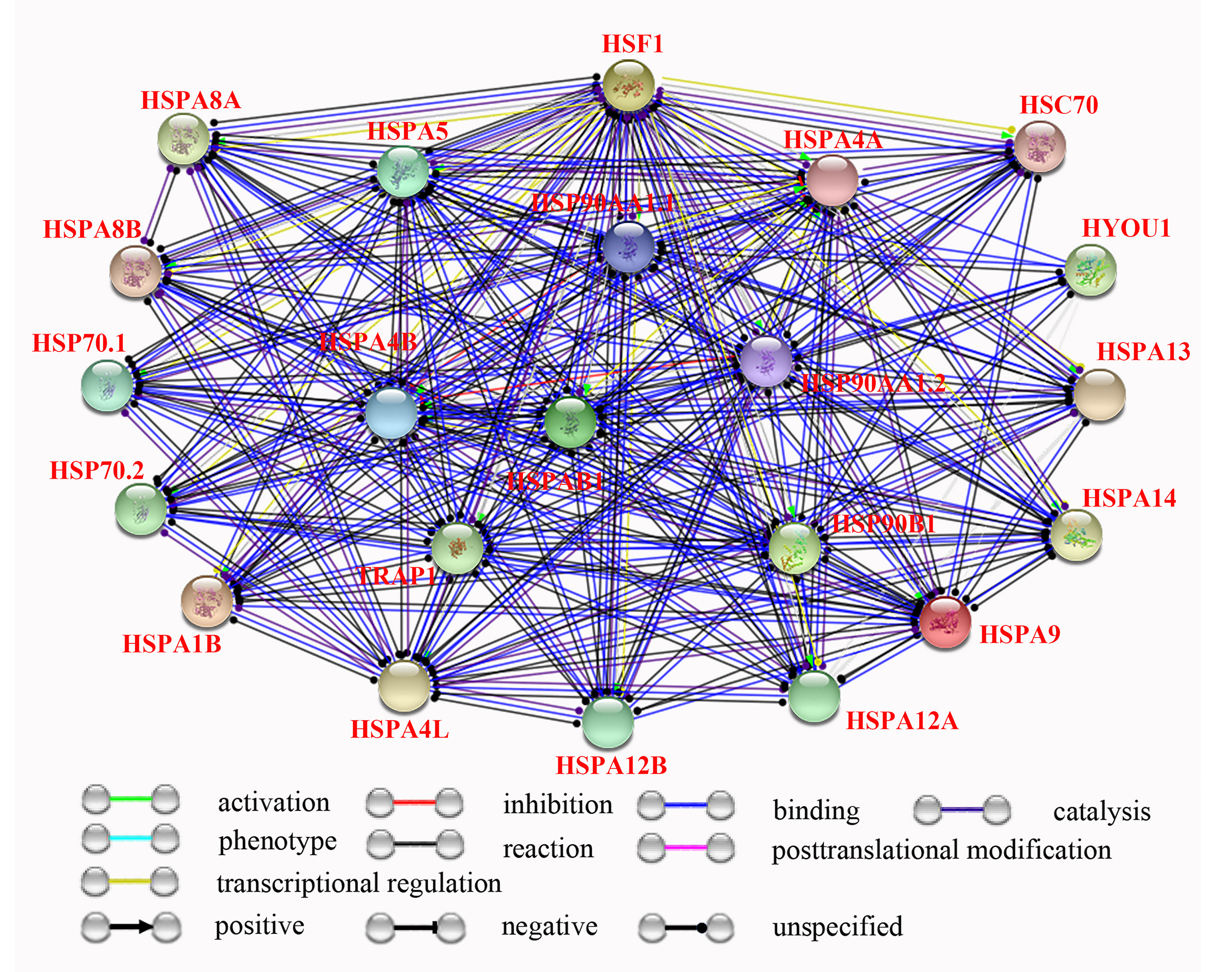

Supplement: Supplementary Figure 2 — Predicted protein-protein interaction network of HSF1 and HSPs in spotted seabass. This network was predicted by the online STRING database. The balls represented the gene nodes, and the connecting lines and arrowheads indicated the interactions. HSP, heat shock proteins; HSF, heat shock factor. [file Image_2.JPEG]
