# Supplementary material for: HSP90 and HSP70 Families in Lateolabrax maculatus: Genome-Wide Identification, Molecular Characterization, and Expression Profiles in Response to Various Environmental Stressors
Source: Front Physiol. 2021 Nov 22;12:784803. doi: 10.3389/fphys.2021.784803 (PMC8646100; doi:10.3389/fphys.2021.784803)
Supplement: Supplementary file 3 [file Data_Sheet_1.docx]

**Supplementary Table 1 List of accession numbers of reference *HSP90* and *HSP70*.**

| **Gene name** | **Accession numbers** | **Species Name** |
| --- | --- | --- |
| HSP90AA1 | ENSP00000216281 | human |
| HSP90AB1 | ENSP00000360709 | human |
| HSP90B1 | ENSP00000299767 | human |
| TRAP1 | ENSP00000246957 | human |

| **Gene name** | **Accession numbers** | **Species Name** |
| --- | --- | --- |
| *Hsp90aa1* | ENSMUSP00000091921 | mouse |
| *Hsp90ab1* | ENSMUSP00000024739 | mouse |
| *Hsp90b1* | ENSMUSP00000020238 | mouse |
| *Trap1* | ENSMUSP00000006137 | mouse |

| **Gene name** | **Accession numbers** | **Species Name** |
| --- | --- | --- |
| *Hsp90aa1* | NP_001103255.1 | chicken |
| *Hsp90ab1* | ENSGALP00000016523 | chicken |
| *Hsp90b1* | ENSGALP00000020744 | chicken |
| *Trap1* | ENSGALP00000012445 | chicken |

| **Gene name** | **Accession numbers** | **Species Name** |
| --- | --- | --- |
| *Hsp90aa1.1* | NP_001016282.1 | tropical clawed frog |
| *Hsp90aa1.2* | NP_001072765.1 | tropical clawed frog |
| *Hsp90ab1* | NP_001025655.1 | tropical clawed frog |
| *Hsp90b1* | NP_001084280.1 | tropical clawed frog |
| *Trap1* | ENSXETP00000058756 | tropical clawed frog |

| **Gene name** | **Accession numbers** | **Species Name** |
| --- | --- | --- |
| *hsp90aa1.1* | ENSDARP00000022302 | zebrafish |
| *hsp90aa1.2* | ENSDARP00000026065 | zebrafish |
| *hsp90ab1* | ENSDARP00000014978 | zebrafish |
| *hsp90b1* | ENSDARP00000013441 | zebrafish |
| *trap1* | ENSDARP00000107323 | zebrafish |

| **Gene name** | **Accession numbers** | **Species Name** |
| --- | --- | --- |
| *hsp90aa1* | XP_015205873.1 | spotted gar |
| *hsp90ab1* | XP_006625986 | spotted gar |
| *hsp90b1* | ENSLOCG00000015012 | spotted gar |
| *trap1* | ENSLOCG00000002737 | spotted gar |

| **Gene name** | **Accession numbers** | **Species Name** |
| --- | --- | --- |
| *hsp90aa1* | ENSAMXG00000020572 | Mexican tetra |
| *hsp90ab1* | ENSAMXG00000024526 | Mexican tetra |
| *hsp90b1* | ENSAMXG00000010077 | Mexican tetra |
| *trap1* | ENSAMXG00000019702 | Mexican tetra |

| **Gene name** | **Accession numbers** | **Species Name** |
| --- | --- | --- |
| *hsp90aa1.1* | ENSCSEG00000000594 | tongue sole |
| *hsp90aa1.2* | ENSCSEG00000000515 | tongue sole |
| *hsp90ab1* | ENSCSEG00000020742 | tongue sole |
| *hsp90b1* | ENSCSEG00000008269 | tongue sole |
| *trap1* | XP_008328085 | tongue sole |

| **Gene name** | **Accession numbers** | **Species Name** |
| --- | --- | --- |
| *hsp90ab1* | ENSSSAG00000040057 | Atlantic salmon |
| *hsp90b1* | ENSSSAG00000070462 | Atlantic salmon |
| *trap1* | XP_014058182 | Atlantic salmon |

| **Gene name** | **Accession numbers** | **Species Name** |
| --- | --- | --- |
| *Hsp90aa1.1* | ENSIPUT00000034371 | channel catfish |
| *Hsp90aa1.2* | ENSIPUT00000034376 | channel catfish |
| *hsp90ab1* | ENSIPUG00000021071 | channel catfish |
| *hsp90b1* | ENSIPUG00000019031 | channel catfish |
| *trap1* | ENSIPUG00000005143 | channel catfish |

| **Gene name** | **Accession numbers** | **Species Name** |
| --- | --- | --- |
| *hsp90aa1.1* | ENSONIP00000001042 | Nile tilapia |
| *hsp90aa1.2* | ENSONIP00000001024 | Nile tilapia |
| *hsp90ab1* | ENSONIP00000007472 | Nile tilapia |
| *hsp90b1* | ENSONIP00000010722 | Nile tilapia |
| *trap1* | ENSONIP00000006501 | Nile tilapia |

| **Gene name** | **Accession numbers** | **Species Name** |
| --- | --- | --- |
| *hsp90aa1* | ENSLCRG00005000648 | large yellow croaker |
| *hsp90ab1* | ENSLCRG00005014951 | large yellow croaker |
| *hsp90b1* | ENSLCRG00005019706 | large yellow croaker |
| *trap1* | ENSLCRG00005003221 | large yellow croaker |

| **Gene name** | **Accession numbers** | **Species Name** |
| --- | --- | --- |
| *hsp90aa1.1* | ENSTRUP00000031409 | fugu |
| *hsp90aa1.2* | ENSTRUP00000031536 | fugu |
| *hsp90ab1* | XP_003971791.1 | fugu |
| *hsp90b1* | ENSTRUP00000043499 | fugu |
| *trap1* | ENSTRUP00000010455 | fugu |

| **Gene name** | **Accession numbers** | **Species Name** |
| --- | --- | --- |
| *hsp90aa1.1* | ENSORLP00000021928 | medaka |
| *hsp90aa1.2* | ENSORLP00000021939 | medaka |
| *hsp90ab1* | ENSORLP00000014900 | medaka |
| *hsp90b1* | ENSORLP00000005467 | medaka |
| *trap1* | ENSORLP00000010499 | medaka |

| **Gene name** | **Accession numbers** | **Species Name** |
| --- | --- | --- |
| HSPA1A | NP_005336.3 | human |
| HSPA1B | NP_005337.2 | human |
| HSPA1L | NP_005518.3 | human |
| HSPA2 | NP_068814.2 | human |
| HSPA4 | NP_002145.3 | human |
| HSPA4L | NP_055093.2 | human |
| HSPA5 | NP_005338.1 | human |
| HSPA6 | NP_002146.2 | human |
| HSPA7 | UniProtKB/Swiss-Prot: P48741.2 | human |
| HSPA8 | NP_006588.1 | human |
| HSPA9 | NP_004125.3 | human |
| HSPA12A | NP_079291.2 | human |
| HSPA12B | NP_443202.3 | human |
| HSPA13 | NP_008879.3 | human |
| HSPA14 | NP_057383.2 | human |
| HSPH1 | NP_006635.2 | human |
| HYOU1 | NP_001124463.1 | human |

| **Gene name** | **Accession numbers** | **Species Name** |
| --- | --- | --- |
| Hspa1a | NP_034609.2 | Mouse |
| Hspa1b | NP_034608.2 | Mouse |
| Hspa1l | NP_038586.2 | Mouse |
| Hspa2 | NP_001002012.1 | Mouse |
| Hspa4 | NP_032326.3 | Mouse |
| Hspa4l | NP_035150.3 | Mouse |
| Hspa5 | NP_001156906.1 | Mouse |
| hspa8 | NP_112442.2 | Mouse |
| Hspa9 | NP_034611.2 | Mouse |
| Hspa12a | NP_780408.1 | Mouse |
| Hspa12b | NP_082582.1 | Mouse |
| Hspa13 | NP_084477.1 | Mouse |
| Hspa14 | NP_056580.2 | Mouse |
| Hsph1 | NP_038587.2 | Mouse |
| Hyou1 | NP_067370.3 | Mouse |
|  |  |  |
| **Gene name** | **Accession numbers** | **Species Name** |
| Hspa2 | NP_001006686.1 | chicken |
| Hspa4 | XP_003642142.1 | chicken |
| Hspa4l | NP_001012594.1 | chicken |
| Hspa5 | NP_990822.1 | chicken |
| Hspa8 | NP_990334.1 | chicken |
| Hspa9 | NP_001006147.1 | chicken |
| Hspa12a | XP_421779.3 | chicken |
| Hspa12b | XP_001233402.2 | chicken |
| Hspa13 | NP_001025964.2 | chicken |
| Hspa14 | XP_416996.3 | chicken |
| Hsph1 | NP_001153170.1 | chicken |
| Hyou1 | NP_001006588.1 | chicken |

| **Gene name** | **Accession numbers** | **Species Name** |
| --- | --- | --- |
| hspa1a | ENSXETT00000071925 | Tropical clawed frog |
| hsp70 | XP_002937685 | Tropical clawed frog |
| hsp70.1l | NP_001072429 | Tropical clawed frog |
| hspa1b | ENSXETG00000041157 | Tropical clawed frog |
| hspa4 | NP_989252.1 | Tropical clawed frog |
| hspa4-201 | ENSXETT00000092584 | Tropical clawed frog |
| hspa4-202 | ENSXETT00000102281 | Tropical clawed frog |
| hspa5 | ENSXETG00000016838 | Tropical clawed frog |
| hspa8a | ENSXETG00000033440 | Tropical clawed frog |
| hspa8b | ENSXETG00000013038 | Tropical clawed frog |
| hsc70 | XP_002937574 | Tropical clawed frog |
| hspa9 | ENSXETG00000008404 | Tropical clawed frog |
| hsp12a | ENSXETG00000014317 | Tropical clawed frog |
| hsp12b | ENSXETG00000012748 | Tropical clawed frog |
| hspa13 | ENSXETT00000061634 | Tropical clawed frog |
| hspa14 | ENSXETG00000009112 | Tropical clawed frog |
| hsph1 | ENSXETG00000019175 | Tropical clawed frog |
| hyou1 | ENSXETG00000010075 | Tropical clawed frog |

| **Gene name** | **Accession numbers** | **Species Name** |
| --- | --- | --- |
| *hsp70.3* | NP_571472.1 | zebrafish |
| *hsp70.2* | XP_003198158.1 | zebrafish |
| *hsp70* | [ENSDARP00000109199](http://useast.ensembl.org/Danio_rerio/Transcript/ProteinSummary?db=core;g=ENSDARG00000029688;r=3:26912773-26922329;t=ENSDART00000124762) | zebrafish |
| *hspa1b* | NP_001093532.1 | zebrafish |
| *hsp70l* | NP_001107061.1 | zebrafish |
| *hspa4a* | NP_999881.1 | zebrafish |
| *hspa4b* | NP_956151.1 | zebrafish |
| *hspa4l* | XP_690505.2 | zebrafish |
| *hspa5* | NP_998223.1 | zebrafish |
| *hspa8a* | NP_001103873.1 | zebrafish |
| *hspa8b* | NP_001186941.1 | zebrafish |
| *hsc70* | [NP_956908.1](http://www.ncbi.nlm.nih.gov/protein/NP_956908.1) | zebrafish |
| *hspa9* | NP_958483.2 | zebrafish |
| *hsp12a.1* | NP_001038342.1 | zebrafish |
| *hsp12a.2* | XP_003198604.1 | zebrafish |
| *hsp12a.3* | NP_001038346.2 | zebrafish |
| *hspa13* | NP_001082948.1 | zebrafish |
| *hspa14* | NP_001038541.1 | zebrafish |
| *hsph1* | XP_001919957.1 | zebrafish |
| *hyou1* | NP_997868.1 | zebrafish |

| **Gene name** | **Accession numbers** | **Species Name** |
| --- | --- | --- |
| *hspa4l* | XP_015200454 | spotted gar |
| *hspa4* | XP_006631871 | spotted gar |
| *hspa12a.2* | ENSLOCG00000009586 | spotted gar |
| *hspa12b* | ENSLOCG00000001328 | spotted gar |
| *hspa12bl* | XP_015194949 | spotted gar |
| *hspa13* | XP_006627784 | spotted gar |
| *hspa5* | XP_006640761 | spotted gar |
| *hspa14* | XP_015208682.1 | spotted gar |
| *hspa9* | ENSLOCG00000012077 | spotted gar |
| *hsph1* | ENSLOCG00000000380 | spotted gar |
| *hsc70* | XP_015193124.1 | spotted gar |
| *hsp70l* | XP_015194712.1 | spotted gar |
| *hyou1* | XP_015193209.1 | spotted gar |

| **Gene name** | **Accession numbers** | **Species Name** |
| --- | --- | --- |
| *hsp70* | XP_022526766.1 | Mexican tetra |
| *hspa1b* | ENSAMXG00000026104 | Mexican tetra |
| *hspa4a* | ENSAMXG00000020109 | Mexican tetra |
| *hspa4b* | ENSAMXG00000011616 | Mexican tetra |
| *hspa4l* | XP_007258636.2 | Mexican tetra |
| *hspa5* | ENSAMXG00000009012 | Mexican tetra |
| *hspa8* | ENSAMXG00000003027 | Mexican tetra |
| *hspa8b* | ENSAMXG00000004386 | Mexican tetra |
| *hsc70l* | XP_022530443.1 | Mexican tetra |
| *hspa9* | ENSAMXG00000002363 | Mexican tetra |
| *hsp12a* | ENSAMXG00000011627 | Mexican tetra |
| *hsp12b* | ENSAMXG00000017515 | Mexican tetra |
| *hspa13* | XP_022537466.1 | Mexican tetra |
| *hspa14* | ENSAMXG00000009721 | Mexican tetra |
| *hsph1* | ENSAMXG00000014428 | Mexican tetra |
| *hyou1* | XP_022537074.1 | Mexican tetra |

| **Gene name** | **Accession numbers** | **Species Name** |
| --- | --- | --- |
| *hsp70* | AJP36131.1 | tongue sole |
| *hsp70l* | XP_008307673.1 | tongue sole |
| *hspa4l.2* | XP_024912926 | tongue sole |
| *hspa4l* | XP_008306103.1 | tongue sole |
| *hspa5* | ENSCSEG00000017610 | tongue sole |
| *hspa8* | ENSCSEG00000019608 | tongue sole |
| *hspa8-201* | ENSCSET00000031016 | tongue sole |
| *hsc70* | XP_008330837.1 | tongue sole |
| *hspa9* | ENSCSEG00000001908 | tongue sole |
| *hsp12a* | ENSCSEG00000018156 | tongue sole |
| *hsp12b* | ENSCSEG00000016674 | tongue sole |
| *hspa13* | XP_00830676 | tongue sole |
| *hspa14* | XP_008314207 | tongue sole |
| *hsph1* | ENSCSEG00000012129 | tongue sole |
| *hyou1* | XP_008330828.1 | tongue sole |

| **Gene name** | **Accession numbers** | **Species Name** |
| --- | --- | --- |
| *Hsp70.3* | ACH70704 | Atlantic salmon |
| *Hspa5l* | ENSSSAG00000042766 | Atlantic salmon |
| *Hspa13l* | ENSSSAG00000032942 | Atlantic salmon |
| *Hspa9l* | ENSSSAG00000047960 | Atlantic salmon |
| *Hyou1* | ENSSSAG00000070523 | Atlantic salmon |
| *Hspa14* | XP_014043218.1 | Atlantic salmon |
| *Hspa12bl* | ENSSSAG00000042868 | Atlantic salmon |
| *Hspa4l* | ENSSSAG00000015877 | Atlantic salmon |
| *Hspa4l.1* | XP_014044105.1 | Atlantic salmon |
| *Hspa4l.2* | XP_014066250.1 | Atlantic salmon |
| *Hspa4l.3* | XP_014066251.1 | Atlantic salmon |
| *Hspa12al.1* | ENSSSAG00000075965 | Atlantic salmon |
| *Hspa12al.2* | ENSSSAG00000001820 | Atlantic salmon |
| *Hsc70* | NP_001135156 | Atlantic salmon |
| *Hspa8* | ENSSSAG00000049191 | Atlantic salmon |
| *Hsp70* | ENSSSAG00000068931 | Atlantic salmon |
| *Hsp70l* | ENSSSAG00000067344 | Atlantic salmon |

| **Gene name** | **Accession numbers** | **Species Name** |
| --- | --- | --- |
| *hsp70.2* | KT961621 | channel catfish |
| *hsp70.3* | JT412120.1 | channel catfish |
| *hspa4a* | JT406739.1 | channel catfish |
| *hspa4b* | JT407108.1 | channel catfish |
| *hspa4l* | JT407194.1 | channel catfish |
| *hspa5* | JT415554.1 | channel catfish |
| *hspa8a.1* | JT315838.1 | channel catfish |
| *hspa8a.2* | NP_001187202.1 | channel catfish |
| *hspa8b* | JT418846.1 | channel catfish |
| *hspa9* | JT408761.1 | channel catfish |
| *hspa12a* | KT961622 | channel catfish |
| *hspa12b* | JT411717.1 | channel catfish |
| *hspa13* | JT413763.1 | channel catfish |
| *hspa14* | JT406759.1 | channel catfish |
| *hyou1* | JT411259.1 | channel catfish |
| *hsc70* | JT408276.1 | channel catfish |

| **Gene name** | **Accession numbers** | **Species Name** |
| --- | --- | --- |
| *hsp70.3* | xp_003442504.1 | Nile tilapia |
| *hspa1b* | xp_003444871.1 | Nile tilapia |
| *hspa8a* | xp_003448938.1 | Nile tilapia |
| *hsc70* | xp_003454400.1 | Nile tilapia |
| *hspa8b* | xp_003455104.1 | Nile tilapia |
| *hspa4l* | xp_003453147.1 | Nile tilapia |
| *hspa5a* | xp_005470418.1 | Nile tilapia |
| *hspa5b* | xp_003459659.1 | Nile tilapia |
| *hspa9* | xp_003459471.1 | Nile tilapia |
| *hspa12a* | xp_003457416.1 | Nile tilapia |
| *hspa12b* | xp_003452414.1 | Nile tilapia |
| *hspa13* | xp_003441638.1 | Nile tilapia |
| *hspa14* | xp_003455685.1 | Nile tilapia |
| *hyou1l* | xp_003448981.1 | Nile tilapia |

| **Gene name** | **Accession numbers** | **Species Name** |
| --- | --- | --- |
| *hsp70* | XP_010753364.1 | large yellow croaker |
| *hspa1b* | XP_010738994.1 | large yellow croaker |
| *hspa4a* | XP_010727629.1 | large yellow croaker |
| *hspa4b* | XP_010753054.2 | large yellow croaker |
| *hspa4l.1* | XP_019132630.1 | large yellow croaker |
| *hspa4l.2* | XP_019135064.1 | large yellow croaker |
| *hspa5.1* | XP_010737097.1 | large yellow croaker |
| *hspa5.2* | XP_019129638.1 | large yellow croaker |
| *hspa8a* | XP_019135026.1 | large yellow croaker |
| *hspa8b* | XP_019126385.1 | large yellow croaker |
| *hsc70* | XP_010745868.1 | large yellow croaker |
| *hspa9* | XP_010732361.2 | large yellow croaker |
| *hspa12a* | XP_019113459.1 | large yellow croaker |
| *hspa12b* | XP_019111192.1 | large yellow croaker |
| *hspa13* | XP_010755439.1 | large yellow croaker |
| *hspa14* | XP_019121868.1 | large yellow croaker |
| *hyou1* | XP_019135047.1 | large yellow croaker |

| **Gene name** | **Accession numbers** | **Species Name** |
| --- | --- | --- |
| *hsp70.3* | XP_003964983.1 | fugu |
| *hspa1b* | XP_003963154.1 | fugu |
| *hspa2* | [ENSTRUT00000005983](http://asia.ensembl.org/Takifugu_rubripes/Transcript/Summary?db=core;g=ENSTRUG00000002565;r=scaffold_248:8531-10608;t=ENSTRUT00000005983) | fugu |
| *hspa8* | XP_003977939.1 | fugu |
| *hsc70* | XP_003966054.1 | fugu |
| *hspa5* | XP_003965205.1 | fugu |
| *hspa9* | XP_003977088.1 | fugu |
| *hspa13* | XP_003968291.1 | fugu |
| *hspa4a* | [ENSTRUT00000021036](http://asia.ensembl.org/Takifugu_rubripes/Transcript/Summary?db=core;g=ENSTRUG00000008366;r=scaffold_82:692563-700490;t=ENSTRUT00000021036) | fugu |
| *hspa4b* | [ENSTRUT00000016139](http://asia.ensembl.org/Takifugu_rubripes/Transcript/Summary?db=core;g=ENSTRUG00000006546;r=scaffold_126:588882-594764;t=ENSTRUT00000016139) | fugu |
| *hspa12a* | [ENSTRUT00000009556](http://asia.ensembl.org/Takifugu_rubripes/Transcript/Summary?db=core;g=ENSTRUG00000004009;r=scaffold_599:33404-37031;t=ENSTRUT00000009556) | fugu |
| *hspa12b* | [ENSTRUT00000006941](http://asia.ensembl.org/Takifugu_rubripes/Transcript/Summary?db=core;g=ENSTRUG00000002957;r=scaffold_315:19527-27736;t=ENSTRUT00000006941) | fugu |
| *hspa14* | [ENSTRUT00000031936](http://asia.ensembl.org/Takifugu_rubripes/Transcript/Summary?db=core;g=ENSTRUG00000012564;r=scaffold_171:552744-556410;t=ENSTRUT00000031936) | fugu |
| *hyou1* | XP_003977948.1 | fugu |

| **Gene name** | **Accession numbers** | **Species Name** |
| --- | --- | --- |
| *hsp70.3* | XP_004071143.1 | medaka |
| *hspa1b* | NP_001098384.1 | medaka |
| *hsc70* | NP_001098385.1 | medaka |
| *hsc70.2* | XP_004075396.1 | medaka |
| *hspa8a* | NP_001098270.1 | medaka |
| *hspa4a* | [ENSORLP00000001795](http://asia.ensembl.org/Oryzias_latipes/Transcript/ProteinSummary?db=core;g=ENSORLG00000001448;r=14:5585364-5596612;t=ENSORLT00000001796) | medaka |
| *hspa4b* | [ENSORLP00000007499](http://asia.ensembl.org/Oryzias_latipes/Transcript/ProteinSummary?db=core;g=ENSORLG00000005958;r=10:13576750-13586189;t=ENSORLT00000007500) | medaka |
| *hspa4l* | XP_004082341.1 | medaka |
| *hspa5l* | XP_004074796.1 | medaka |
| *hspa9* | [ENSORLP00000013340](http://asia.ensembl.org/Oryzias_latipes/Transcript/ProteinSummary?db=core;g=ENSORLG00000010637;r=10:24154105-24160954;t=ENSORLT00000013341) | medaka |
| *hspa12a* | [ENSORLP00000001447](http://asia.ensembl.org/Oryzias_latipes/Transcript/ProteinSummary?db=core;g=ENSORLG00000001174;r=15:3764907-3799543;t=ENSORLT00000001448) | medaka |
| *hspa12b* | [ENSORLP00000007349](http://asia.ensembl.org/Oryzias_latipes/Transcript/ProteinSummary?db=core;g=ENSORLG00000005839;r=18:12654023-12673045;t=ENSORLT00000007350) | medaka |
| *hspa13l* | XP_004075919.1 | medaka |
| *hspa14* | [ENSORLP00000015785](http://asia.ensembl.org/Oryzias_latipes/Transcript/ProteinSummary?db=core;g=ENSORLG00000012603;r=23:10901561-10912448;t=ENSORLT00000015786) | medaka |
| *hyou1l* | XP_004084567.1 | medaka |

**Supplementary Table 2 Information regarding the STRING search results of the interaction networks of the predicted HSF1 and HSP proteins.**

| **Query Sequence in Spotted Sea Bass** | **STRING Protein in *Danio rerio*** | **Annotation** | **Identity** | **Bitscore** |
| --- | --- | --- | --- | --- |
| hsp90aa1.1 | hsp90aa1.1 | Heat shock protein HSP 90-alpha 1 | 91% | 1051.2 |
| hsp90aa1.2 | hsp90aa1.2 | Heat shock protein 90, alpha (cytosolic), class A member 1, tandem duplicate 2 | 107% | 1151.3 |
| hsp90ab1 | hsp90ab1 | Heat shock protein HSP 90-beta | 101% | 1125.2 |
| hsp90b1 | hsp90b1 | Heat shock protein 90, beta (grp94), member 1 | 88% | 1135.6 |
| trap1 | trap1 | TNF receptor-associated protein 1 | 84% | 1166 |
| hspa8a.1 | hspa8 | Heat shock cognate 71 kDa protein | 95% | 991.9 |
| hspa8a.2 | hspa8 | Heat shock cognate 71 kDa protein | 92% | 1029.6 |
| hspa8b | ENSDARG00000037403 | Si:dkey-4p15.3; Heat shock 70kDa protein 8 | 96% | 1028.1 |
| hsc70 | hsc70 | Heat shock cognate 70; Belongs to the heat shock protein 70 family | 95% | 1147.1 |
| hspa1b | ENSDARG00000056210 | Si:ch211-199o1.2; Belongs to the heat shock protein 70 family | 89% | 1122.8 |
| hsp70.1 | zgc:174006 | Heat shock cognate 70-kd protein, tandem duplicate 1; MCM5 minichromosome maintenance deficient 5 (S. cerevisiae); Belongs to the heat shock protein 70 family | 87% | 1063.5 |
| hsp70.2 | hsp70.2 | Heat shock cognate 70-kd protein, tandem duplicate 2 | 88% | 1106.3 |
| hspa5 | hspa5 | Heat shock protein 5; Belongs to the heat shock protein 70 family | 96% | 1176 |
| hspa9 | hspa9 | Heat shock protein 9; Belongs to the heat shock protein 70 family | 90% | 1156.4 |
| hspa13 | hspa13 | Heat shock protein 13; Belongs to the heat shock protein 70 family | 82% | 726.9 |
| hyou1 | hyou1 | Hypoxia up-regulated protein 1 | 92% | 1238.4 |
| hspa4a | hspa4a | Heat shock protein 4a; Belongs to the heat shock protein 70 family | 86% | 1236.1 |
| hspa4b | hspa4b | Heat shock protein 4b; Belongs to the heat shock protein 70 family | 91% | 1109 |
| hspa4l | HSPA4L | Heat shock 70kDa protein 4-like | 81% | 1217.6 |
| hspa14 | hspa14 | Heat shock 70 kDa protein 14 | 74% | 731.5 |
| hspa12a | hspa12a | Heat shock protein 12A | 91% | 1231.9 |
| hspa12b | hspa12b | Heat shock protein 12B | 85% | 1185.2 |

**Supplementary Table 3 Expression fold change of *HSP90s* and *HSP70s* in gills of spotted sea bass in response to alkalinity challenges.**

| Gene name | H12 vs H0 | p_value | H24 vs H0 | p_value | H72 vs H0 | p_value |
| --- | --- | --- | --- | --- | --- | --- |
| *hsp90aa1.1* | -1.19 | 1.0000 | 1.42 | 1.0000 | -1.01 | 1.0000 |
| *hsp90aa1.2* | -1.14 | 0.2236 | -1.50 | 0.0002 | -1.69 | 0.0001 |
| *hsp90ab1* | 1.01 | 0.9217 | 1.10 | 0.4570 | 1.19 | 0.1690 |
| *hsp90b1* | 1.01 | 0.0911 | -1.05 | 0.6803 | -1.35 | 0.0351 |
| *trap1* | 1.18 | 0.9515 | -1.04 | 0.6654 | -1.23 | 0.0078 |
| *hspa1b* | -2.06 | 1.0000 | -2.64 | 1.0000 | -1.87 | 1.0000 |
| *hsp70.1* | -1.01 | 0.9714 | -1.01 | 0.9604 | -1.43 | 0.2240 |
| *hsp70.2* | -1.09 | 0.6980 | -1.23 | 0.3620 | -1.60 | 0.0471 |
| *hspa8a.2* | 1.01 | 0.9553 | 1.03 | 0.7767 | 1.04 | 0.6866 |
| *hspa8a.1* | -1.47 | 0.0001 | -1.51 | 0.0001 | -1.58 | 0.0001 |
| *hsc70* | -1.62 | 0.0682 | -1.69 | 0.0463 | -1.09 | 0.7191 |
| *hspa8b* | 1.03 | 0.7327 | -1.19 | 0.0720 | -1.03 | 0.7524 |
| *hspa13* | -1.62 | 0.0001 | -1.70 | 0.0001 | -3.06 | 0.0001 |
| *hspa4b* | -1.03 | 0.7806 | -1.02 | 0.7898 | -1.15 | 0.1337 |
| *hspa4l* | -1.26 | 0.2143 | -1.14 | 0.4845 | -1.30 | 0.1657 |
| *hspa4a* | 1.01 | 0.8788 | -1.12 | 0.2250 | -1.25 | 0.0185 |
| *hspa14* | -1.13 | 0.3106 | -1.68 | 0.0001 | -2.04 | 0.0001 |
| *hspa12a* | 1.11 | 0.3973 | 1.38 | 0.0089 | 1.67 | 0.0001 |
| *hspa12a* | 1.18 | 0.1212 | 1.16 | 0.1656 | 1.42 | 0.0015 |
| *hspa5* | -1.08 | 0.4238 | -1.79 | 0.0001 | -2.54 | 0.0001 |
| *hspa9* | 0.00 | 1.0000 | 0.00 | 1.0000 | 0.00 | 1.0000 |
| *hyou1* | -1.09 | 0.3537 | -1.39 | 0.0002 | -0.72 | 0.0001 |

**Supplementary Table 4 Expression fold change of *HSP90s* and *HSP70s* in gills of spotted sea bass in response to hypoxia challenges.**

| Gene name | H3 vs H0 | p_value | H6 vs H0 | p_value | H12 vs H0 | p_value |
| --- | --- | --- | --- | --- | --- | --- |
| *hsp90aa1.1* | 1.70 | 0.1248 | 1.42 | 0.3116 | 1.95 | 0.1121 |
| *hsp90aa1.2* | -1.01 | 0.9718 | 1.58 | 0.0013 | 3.97 | 0.0000 |
| *hsp90ab1* | -1.23 | 0.0337 | 1.08 | 0.3759 | -1.14 | 0.3367 |
| *hsp90b1* | 1.49 | 0.0011 | 1.20 | 0.1563 | 1.50 | 0.0397 |
| *trap1* | -1.44 | 0.0001 | -1.47 | 0.0005 | -1.23 | 0.1285 |
| *hspa1b* | 0.00 | 1.0000 | 0.00 | 1.0000 | 0.00 | 1.0000 |
| *hsp70.1* | -1.04 | 0.8318 | 1.33 | 0.1505 | 3.15 | 0.0007 |
| *hsp70.2* | -1.02 | 0.9350 | 1.34 | 0.1602 | 3.24 | 0.0009 |
| *hspa8a.2* | -1.19 | 0.1069 | 1.23 | 0.0596 | -1.14 | 0.4332 |
| *hspa8a.1* | -1.16 | 0.1562 | 1.05 | 0.6288 | -1.26 | 0.0724 |
| *hsc70* | 1.60 | 0.1848 | 1.98 | 0.0523 | 2.11 | 0.0791 |
| *hspa8b* | -1.15 | 0.4780 | 1.13 | 0.5156 | 1.59 | 0.0108 |
| *hspa13* | -1.22 | 0.1448 | -1.39 | 0.0125 | -1.27 | 0.1328 |
| *hspa4b* | 1.05 | 0.6441 | 1.18 | 0.0874 | 1.14 | 0.2400 |
| *hspa4l* | 1.01 | 0.9715 | -1.00 | 0.9945 | 1.44 | 0.2259 |
| *hspa4a* | -1.12 | 0.3860 | 1.12 | 0.3977 | 1.79 | 0.0072 |
| *hspa14* | -1.29 | 0.0450 | -1.26 | 0.0394 | -1.09 | 0.5331 |
| *hspa12a* | 1.04 | 0.8143 | -1.30 | 0.2016 | -1.16 | 0.5996 |
| *hspa12a* | 1.75 | 0.0001 | 1.52 | 0.0006 | 2.19 | 0.0000 |
| *hspa5* | 1.09 | 0.6057 | -1.22 | 0.0917 | 1.08 | 0.7352 |
| *hspa9* | 0.00 | 1.0000 | 0.00 | 1.0000 | 0.00 | 1.0000 |
| *hyou1* | 1.07 | 0.6378 | -1.30 | 0.0054 | -1.05 | 0.7270 |
